# Effect of cyclic topology *versus* linear terpolymers on antibacterial activity and biocompatibility: antimicrobial peptide avatars†

**DOI:** 10.1039/d4sc05797j

**Published:** 2024-10-24

**Authors:** Md Aquib, Wenting Yang, Luofeng Yu, Vinod Kumar Kannaujiya, Yuhao Zhang, Peng Li, Andrew Whittaker, Changkui Fu, Cyrille Boyer

**Affiliations:** a Cluster for Advanced Macromolecular Design (CAMD) and Australian Centre for NanoMedicine (ACN), School of Chemical Engineering UNSW Australia Sydney NSW 2052 Australia; b Australian Institute for Bioengineering and Nanotechnology, The University of Queensland St Lucia Queensland 4072 Australia; c Frontiers Science Center for Flexible Electronics (FSCFE), Xi'an Institute of Flexible Electronics (IFE) and Xi'an Institute of Biomedical Materials & Engineering (IBME), Northwestern Polytechnical University 127 West Youyi Road Xi'an 710072 China cboyer@unsw.edu.au changkui.fu@uq.edu.au

## Abstract

Host-defense peptides (HDPs) and their analogs hold significant potential for combating multidrug-resistant (MDR) bacterial infections. However, their clinical use has been hindered by susceptibility to proteases, high production costs, and cytotoxicity towards mammalian cells. Synthetic polymers with diverse topologies and compositions, designed to mimic HDPs, show promise for treating bacterial infections. In this study, we explored the antibacterial activity and biocompatibility of synthetic amphiphilic linear (LPs) and cyclic terpolymers (CPs) containing hydrophobic groups 2-ethylhexyl (E) and 2-phenylethyl (P) at 20% and 30% content. LPs were synthesized *via* RAFT polymerization and then cyclized into CPs through a hetero-Diels–Alder click reaction. The bioactivity of these terpolymers was correlated with their topology (LPs *vs.* CPs) and hydrophobic composition. LPs demonstrated superior antibacterial efficacy compared to CPs against four Gram-negative bacterial strains, with terpolymers containing (P) outperforming those with (E). Increasing the hydrophobicity from 20% to 30% in the terpolymers increased toxicity to both bacterial and mammalian cells. Notably, our terpolymers inhibited the MDR Gram-negative bacterial strain PA37 more effectively than gentamicin and ciprofloxacin. Furthermore, our terpolymers were able to disrupt cell membranes and rapidly eliminate Gram-negative bacteria (99.99% within 15 minutes). Interestingly, CPs exhibited higher hemocompatibility and biocompatibility with mammalian macrophage cells compared to LPs, showcasing a better safety profile (CPs > LPs). These findings underscore the importance of tailoring polymer architectures and optimizing the hydrophilic/hydrophobic balance to address challenges related to toxicity and selectivity in developing antimicrobial polymers.

## Abstract 2

To develop safer and more effective antimicrobial polymers, this study investigates how the structure (linear *vs.* cyclic) and hydrophilic/hydrophobic balance of terpolymers affect their toxicity and selectivity against bacteria.

## Graphical abstract

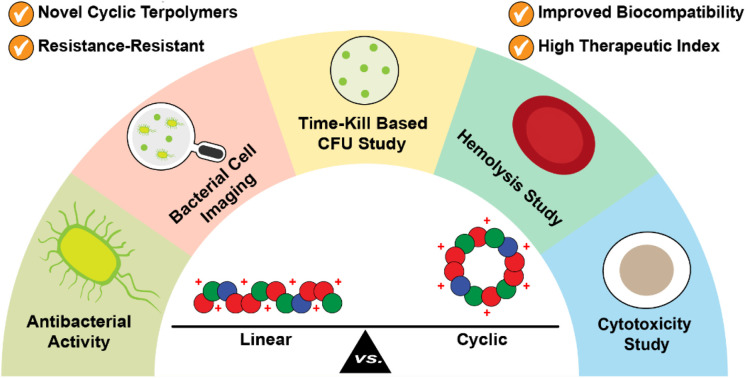

## Introduction

Antimicrobial resistance (AMR) is a pressing global health crisis, recognized by the World Health Organization (WHO) as one of the top 10 global health threats in 2021.^1,2^ While AMR can arise naturally through bacterial evolution, the alarming increase is primarily driven by the misuse of antibiotics in humans, animals, and agriculture,^3,4^ which was recently exacerbated by the COVID-19 pandemic.^5^ The emergence of highly resistant strains, particularly against last-resort antibiotics, underscores the urgent need for novel therapeutic approaches.^3,6,7^ A 2022 WHO report concluded that both existing and developing antibacterials are insufficient to address the growing AMR crisis.^8^ Since 2017, only 12 new antibiotics have been approved,^8^ and offer limited clinical advantages over existing antimicrobial therapies, with most belonging to established antibiotic classes with known resistance mechanisms.^8^ Without innovative solutions, AMR infections are projected to become increasingly difficult to manage, potentially surpassing cancer as a leading cause of death by 2050.^9^

One promising solution is the development of synthetic antimicrobial polymers (APs), which are bio-inspired macromolecules that mimic host defense peptides (HDPs).^10–13^ APs are designated to kill bacteria through membrane disruption, a mechanism believed to be less susceptible to resistance development.^12–17^ Typically composed of cationic and hydrophobic components,^13,18^ APs preferably target negatively charged bacterial cell surfaces,^19,20^ while largely sparing the less negatively charged mammalian cell membranes.^11,12,19,21,22^ However, the inherent presence of positive charges can lead to cytotoxicity and selectivity issues,^23^ limiting their clinical applications.^24^

Therefore, a major focus in AP research is optimizing selectivity by enhancing antimicrobial activity while minimizing non-specific toxicity towards non-bacterial cells.^25,26^ The antibacterial activity and biocompatibility of APs are influenced by various structural components, including the type of cationic groups, monomer sequence, molecular weight dispersity, molecular weight, chemical composition, amphiphilic balance, and topology.^13,27–32^ Recent advances in polymer synthesis, particularly in reversible-deactivation radical polymerization,^33–36^ have allowed researchers to fine-tune these parameters, resulting in the design of macromolecules with enhanced antimicrobial activities and superior biocompatibility.^12,37–41^ Studies have highlighted the importance of polymer topologies, such as bottlebrushes,^42–44^ star,^37,45–47^ hyperbranched,^37,40^ and cyclic architectures,^48–50^ in improving the biocompatibility of APs compared to their linear counterparts.^38,51^ Among the explored polymer topologies, cyclic polymers, characterized by their ring-like structures without chain ends,^52–54^ have garnered sporadic interest in bioapplications.^55,56^ Due to their unique topology, cyclic polymers exhibit reduced hydrodynamic size, enhanced stability, and slower degradation compared to their linear counterparts.^57,58^ These structural differences also influence chain mobility, impacting self-assembly behavior in solution,^55,59^ which can lead to prolonged blood circulation time, greater drug-loading capacity, and improved colloidal stability.^56,60,61^ As a result, cyclic polymers are emerging as attractive materials for bioapplications,^55,56,62^ especially in antibacterial contexts.^48–50^

To date, only a very limited number of studies (three, to our knowledge) have investigated the antimicrobial activity of cyclic polymers.^48–50^ Among them, the work of Duan and colleagues is notable for being the only to report the preparation of cationic cyclic copolymers through the copolymerization of vinyl monomers, specifically 2-(*N*,*N*′-dimethylamino)ethyl methacrylate with a hydrophobic monomer. Their findings revealed that cyclic copolymers exhibited superior antimicrobial efficacy and lower cytotoxicity compared to their linear analogs, largely due to their compact and reduced hydrodynamic volume.^48^ The Alabi's team and Verhaeghe & Bonduelle's team have reported the formation of cyclic oligothioetheramide and peptides and investigated their antimicrobial activities.^49,50^

Building on these pioneering efforts, we decided to prepare for the first time cyclic terpolymers. In this study, we leverage a combination of reversible addition–fragmentation chain transfer polymerization and hetero photo-mediated Diels–Alder click reaction, introduced by Barner–Kowollik's group,^63–67^ to prepare a library of linear and cyclic amphiphilic cationic statistical terpolymers. By systematically investigating the effects of topology (linear *vs.* cyclic), compositions and hydrophobicity, we aim to elucidate the structure–activity relationships governing their antibacterial, hemolytic, and cytotoxic properties, thereby advancing the development of effective APs.

## Results and discussion

### Synthesis and characterization of linear and cyclic terpolymers

**Fig. 1:**
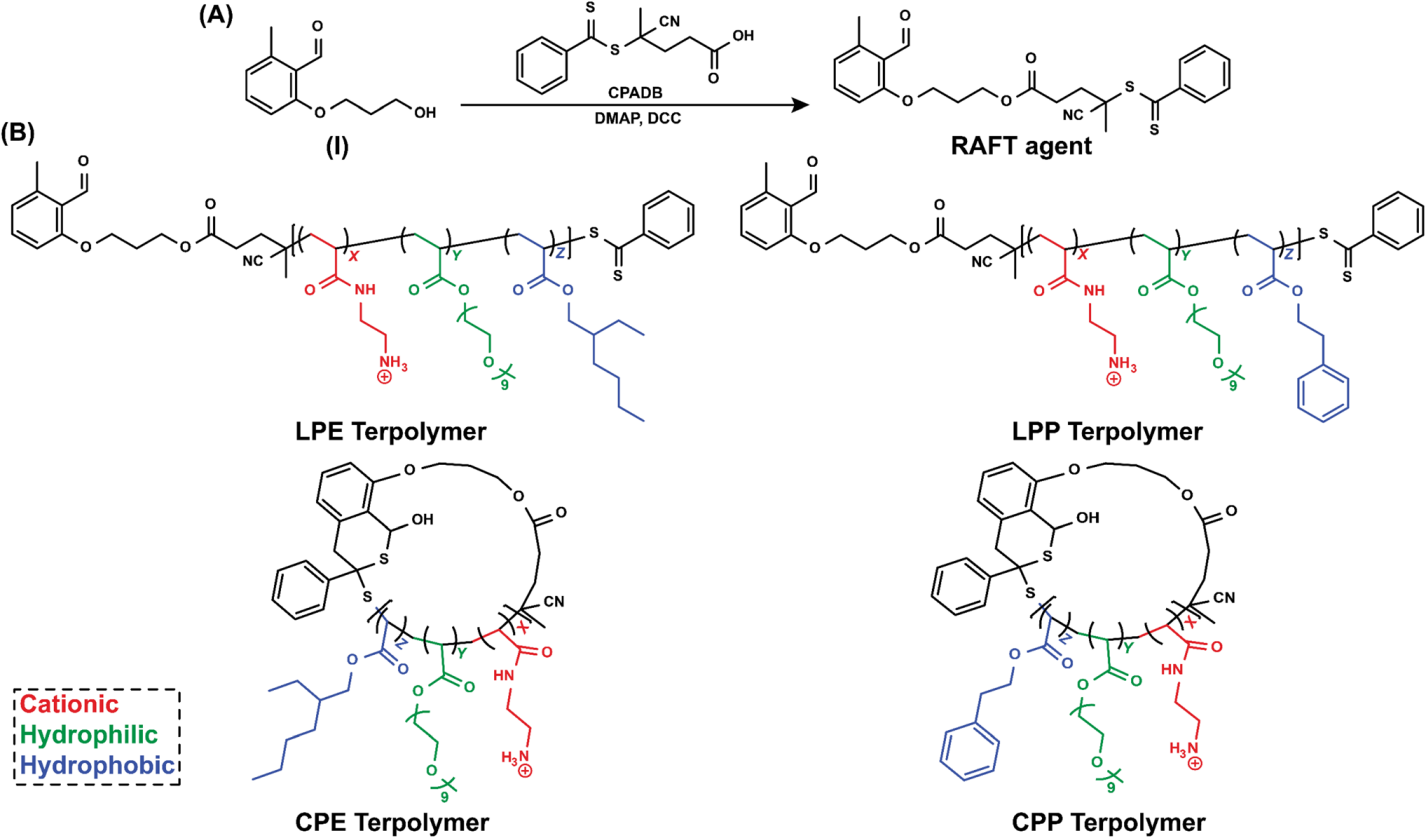
(A) Synthesis scheme of RAFT agent. (B) Chemical structures of amphiphilic LPE, CPE, LPP, and CPP (*X* = percentage of cationic groups, *Y* = percentage of hydrophilic groups, and *Z* = percentage of hydrophobic groups.

In this study, we utilized a reversible addition–fragmentation chain transfer (RAFT) polymerization to synthesize amphiphilic cationic statistical linear terpolymers (LP), which was further cyclized into cyclic terpolymers (CP) using hetero-Diels–Alder click reaction. RAFT polymerization was selected for its control over a wide range of monomers^28,34,37,41,68,69^ and molecular weight, as well as its compatibility with light-induced Diels–Alder cyclization reactions.^63,64,70^ A functional RAFT agent containing a benzaldehyde group for subsequent Diels–Alder cyclization was synthesized by reacting 2-(3-hydroxypropoxy)-6-methylbenzaldehyde (I) with 4-cyano-4-(phenylcarbonothioylthio)pentanoic acid (CPADB) (Fig. 1A).^64^ The successful formation of the RAFT agent was confirmed by ^1^H NMR analysis (ESI (ESI), Fig. S2†) and UV–vis spectroscopy, with the latter revealing a characteristic absorption peak at ∼310 nm attributed to the characteristic π–π* transition of the thiocarbonyl group in the RAFT agent (ESI, Fig. S3†).

Following our previous study,^37^ we then utilized our prepared RAFT agent to synthesize terpolymers with a targeted degree of polymerization (*X*_n_) of 50 (ESI, Fig. S4†). Each terpolymer maintained a constant 50% ratio of the cationic monomer *tert*-butyl (2-acrylamidoethyl) carbamate (Boc-AEAm), while varying the proportion of neutral hydrophilic monomer, polyethylene glycol methyl ether acrylate (PEGMEA) from 20% to 30%. The remaining 30% or 20% of each terpolymer consisted of one of two hydrophobic monomers: either the branched 2-ethylhexyl acrylate (E) or the aromatic 2-phenylethyl acrylate (P) (ESI, Fig. S5–S8†). The resulting polymers were purified by precipitation and divided into two batches. One batch was subjected to Diels–Alder cyclization reaction under 310 nm light, while the other batch was left unreacted. Subsequently, both LPs and CPs underwent Boc deprotection with trifluoroacetic acid (TFA) to generate primary ammonium functionalities. These terpolymers were systematically named according to their topology (LP and CP), hydrophobic monomer types (E or P), and composition (20% or 30% hydrophobic monomer). For example, a linear polymer containing 20% E monomer was designated LPE-20, while its cyclic counterpart was named CPE-20.

### Synthesis and characterization of linear terpolymers

**Fig. 2:**
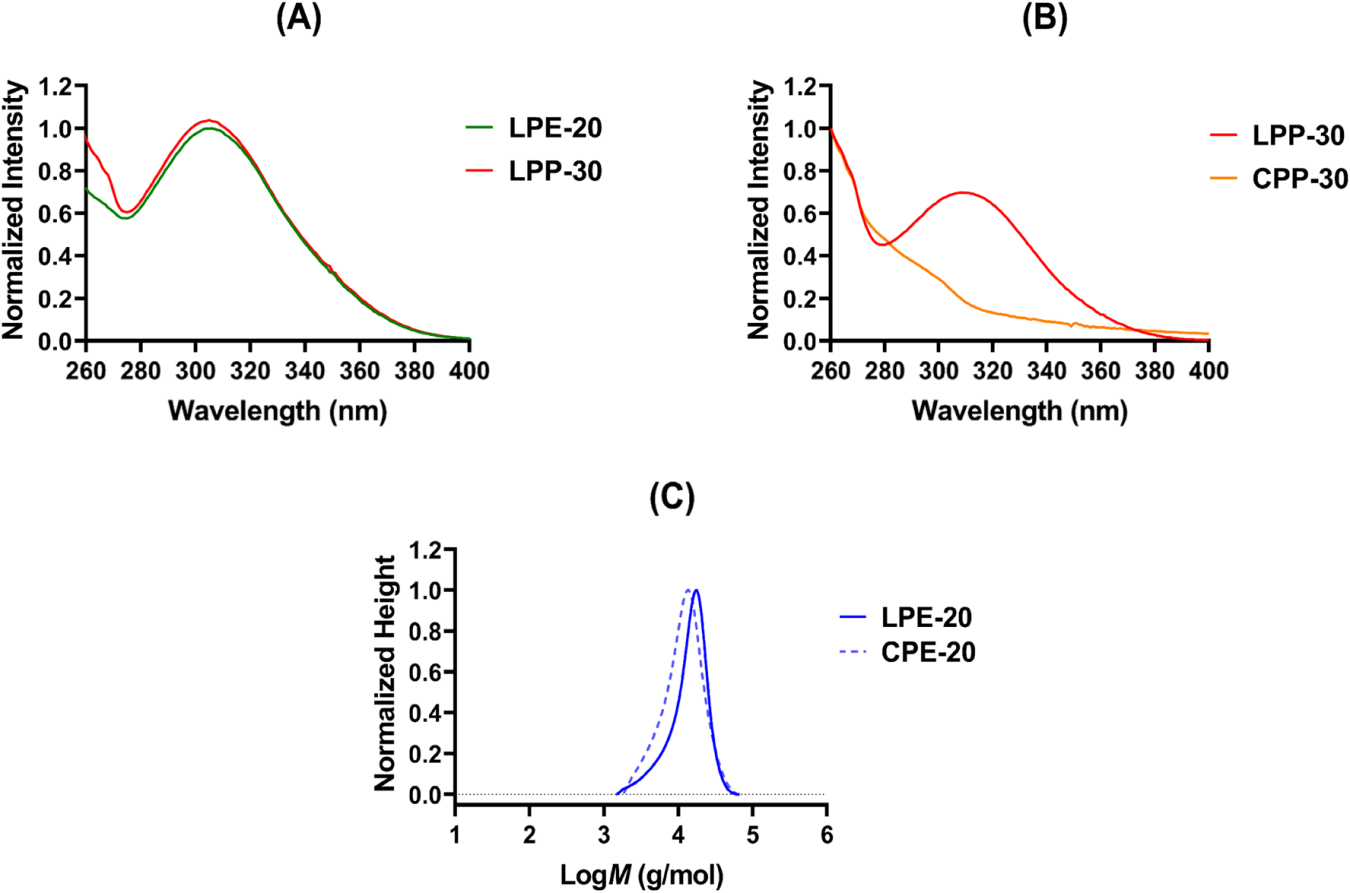
(A) UV-vis spectra of LPs (LPE-20 and LPP-30) in DMSO. (B) UV-vis spectra of Boc-protected LP (LPP-30) and its corresponding CP (CPP-30) in DMSO. (C) MWDs of the Boc-protected LPE-20 and CPE-20 terpolymers obtained by SEC.

The monomer feed ratios for LPE-20, LPE-30, LPP-20, and LPP-30 were confirmed *via*^1^H NMR spectroscopy of crude reactions at *t* = 0 (ESI, Fig. S9 and S14†). After 30 h of polymerization at 70 °C, monomer conversions ranged between 90% and 93% (ESI, Fig. S10 and S15 and Table S1†). The resulting Boc-protected LPs (LPE-20, LPE-30, LPP-20, and LPP-30) showed minimal deviations from the initial feed ratios, as determined by ^1^H NMR spectroscopy (ESI, Fig. S10 and S15†). Notably, ^1^H NMR spectra of the Boc-protected LPs displayed characteristic peaks of the RAFT agent. Signals around 10.55 ppm and 2.46 ppm are attributed to CH-aldehyde group ((a) ESI, Fig. S10 and S15†) and –CH_3_ group ((d and e) ESI, Fig. S10 and S15,† respectively) of orthoquinodimethane moiety, confirming the presence of the RAFT agent.^68,69^ These Boc-protected LPs displayed well-controlled, monomodal molecular weight distributions (MWDs) with dispersity (*Đ*) values ranging from 1.16 to 1.40 (Table 1 and ESI, Fig. S19†), as determined by size exclusion chromatography (SEC). It is important to note that the experimental *M*_n_ determined by SEC deviated from the theoretical and ^1^H NMR-calculated values (Table 1). This discrepancy was attributed to differences in hydrodynamic volume between the PMMA standards and the terpolymers, which was previously reported.^37,71^ Effective removal of Boc-groups by treatment with TFA, followed by purification by precipitation, was verified by the absence of *tert*-butyl protons at *δ* = 1.45 ppm and the presence of a free cationic primary ammonium groups at *δ* ≈ 8.2 ppm (ESI, Fig. S11 and S16†). Importantly, the characteristic peaks of the orthoquinodimethane moiety (CH-aldehyde (a) and –CH_3_ (d, e)) remained visible in the ^1^H NMR spectra of the purified terpolymers (ESI, Fig. S11 and S16†), indicating the stability of the RAFT agent post-TFA treatment. Furthermore, UV-vis spectra of the LPs (LPE-20 and LPP-30), confirmed the characteristic π–π* absorption peak of the thiocarbonyl moiety at approximately 310 nm (Fig. 2A), thus verifying the presence of orthoquinodimethane and dithiobenzoate moieties at the two terminal ends of the terpolymers.^68,69^

**Table 1:** Summary of prepared polymer properties. Polymer compositions (mol%), molecular weights (*M*_n_), dispersity (*Đ*), average hydrodynamic diameter (*D*_h_), zeta potential (*ζ*), and *M*_n,cyclic_/*M*_n,linear_ ratio values were determined by ^1^H NMR, SEC, and DLS analyses

Polymer	Feed ratio cationic : hydrophilic : hydrophobic (mol%)	Polymer composition cationic : hydrophilic : hydrophobic^a^^,^^b^ (mol%)	Targeted *M*_n_ (kg mol^−1^)	*M*_n_ a ^,^ b (kg mol^−1^)	*M*_n_ b ^,^ c (kg mol^−1^)	*Đ* b ^,^ c	*D*_h_ d ^,^ e (nm)	*D*_h_ d ^,^ f (nm)	*ζ* d ^,^ e (mV)	*M*_n, cyclic_/*M*_n, linear_^b^^,^^c^
LPE-20	50 : 30 : 20	52 : 30 : 18	14.8	14.2	12.0	1.36	113.8 ± 8.2	4.3 ± 0.7	56.9 ± 2.4	0.85
CPE-20	50 : 30 : 20	52 : 30 : 18	14.8	14.2	10.2	1.41	76.1 ± 5.2	5.0 ± 0.3	49.4 ± 0.4	
LPE-30	50 : 20 : 30	52 : 20 : 28	13.3	13.5	14.5	1.16	108.8 ± 13.3	3.6 ± 0.8	61.8 ± 0.9	0.82
CPE-30	50 : 20 : 30	52 : 20 : 28	13.3	13.5	12.0	1.38	102.9 ± 7.1	5.3 ± 0.6	57.2 ± 0.2	
LPP-20	50 : 30 : 20	50 : 29 : 21	14.7	16.4	11.1	1.30	97.9 ± 7.1	4.1 ± 0.2	39.9 ± 0.1	1.0
CPP-20	50 : 30 : 20	50 : 29 : 21	14.7	16.4	11.3	1.34	88.5 ± 6.1	6.4 ± 0.4	41.6 ± 0.5	
LPP-30	50 : 20 : 30	49 : 20 : 30	13.2	13.4	11.4	1.40	119.7 ± 8.2	8.0 ± 1.4	58.7 ± 0.7	0.92
CPP-30	50 : 20 : 30	49 : 20 : 30	13.2	13.4	10.6	1.45	106.2 ± 28.6	5.3 ± 1.0	56.3 ± 0.3	

a Determined by ^1^H NMR spectroscopy after polymerization (*t* = 30 h) (calculations available in ESI).

b Based on Boc protected polymers.

c Determined by SEC-DMAc analysis using poly(methyl methacrylate) (PMMA) as the standard.

d Based on Boc deprotected terpolymers.

e Analysis performed in Milli-Q water (DLS).

f Analysis performed in PBS (pH 7.4; DLS). Terpolymers were synthesized with a targeted *X*_n_ of 50.

### Synthesis and characterization of cyclic terpolymers

The Boc-protected linear precursors were subsequently cyclized into Boc-protected CPs *via* a 310 nm light-induced Diels–Alder click reaction. To promote intramolecular cyclization over intermolecular coupling, the reaction was carried out under a highly diluted reaction condition (25 mg L^−1^).^68^ Mild UV irradiation triggered the formation of photoenol groups from the orthoquinodimethane end groups,^68,69^ which then underwent Diels–Alder cycloaddition with the dithioester groups located at the opposite termini of the LPs. This reaction proceeded efficiently at room temperature in the presence of air.^68,69^ Upon 12 hours of irradiation, the resulting Boc-protected CPs were readily isolated by solvent evaporation (ESI, Fig. S4†).

The success of the cyclization reaction and the formation of Boc-protected CPs were confirmed by a combination of ^1^H-NMR, SEC, and UV-vis spectroscopy analyses. The ^1^H-NMR spectra of the Boc-protected CPs indicated the complete disappearance of peaks at around 10.55 and 2.46 ppm (ESI, Fig. S12 and S17†), which correspond to the CH aldehyde group and methyl group of orthoquinodimethane moiety in the Boc-protected linear precursors (ESI, Fig. S10 and S15†).^68,69^ While the proton signal of the newly formed isothiochroman group, expected upon successful cyclization, could not definitively be identified in the ^1^H-NMR spectrum (ESI, Fig. S12 and S17†) due to the complexity of the terpolymer system, the absence of the orthoquinodimethane (–CH_3_ and CH-aldehyde) peaks strongly supports the successful formation of cyclic structures. UV–vis spectroscopy further corroborated the successful cyclization. The characteristic absorption peak around 310 nm, associated with the dithiobenzoate moiety of the linear precursor (Fig. 2B), disappeared in the spectra of the Boc-protected CP (Fig. 2B), confirming the loss of the dithiobenzoate group upon cyclization. Furthermore, a visual change from the initial pink color of the LP to the brown color of the CP was observed after cyclization (ESI, Fig. S20A†).

In addition, SEC analysis confirmed the successful cyclization by revealing the expected shift of MWDs to lower values for the Boc-protected CPs compared to their linear analogues (Fig. 2C and ESI, Fig. S19†). This shift, along with the observed smaller number-average molecular weight (*M*_n_) of Boc-protected CPs compared to their linear counterparts (Table 1), is attributed to the reduced hydrodynamic volume of cyclic polymers.^68,69,72^ The *M*_n,cyclic_/*M*_n,linear_ ratios in our study were calculated and found to be slightly higher than the 0.8 reported in previous publications involving the cyclization of homopolymers.^52,63,68^ However, studies on cyclic copolymers have also shown *M*_n,cyclic_/*M*_n,linear_ ratios greater than 0.8, which was attributed to the specific structure of these copolymers containing different monomers.^48,50^ In addition, minor coupling reactions, likely due to the complex ternary monomer system and steric hindrance from the bulky PEGMEA groups, were observed in the SEC chromatograms (Fig. 2C and ESI, Fig. S19†), resulting in a slight increase in *Đ* (1.34 to 1.45) compared to their linear analogues (Table 1) consistent with other cyclic polymer synthesis study.^72^ Finally, the successful deprotection of Boc-groups from CPs using TFA was confirmed by the absence of *tert*-butyl group at around *δ* = 1.45 ppm and the emergence of cationic primary ammonium groups at *δ* ≈ 8.2 ppm (ESI, Fig. S13 and S18†).

Upon purification, the terpolymers (LPs and CPs) (ESI, Fig. S20B and C†) were dissolved in Milli-Q water and subjected to dynamic light scattering (DLS) analysis. The amphiphilic LPs exhibited average hydrodynamic diameter (*D*_h_) values ranging from 97.9 to 119.7 nm in Milli-Q water (Table 1). In contrast, the CPs consistently displayed smaller average *D*_h_ values, ranging from 76.1 to 106.2 nm (Table 1). This reduction in *D*_h_ for CPs aligns with expectations due to their reduced hydrodynamic volume compared to linear polymers. Zeta potential (*ζ*) measurements for both LPs and CPs revealed positive values ranging from 39.9 to 61.8 mV in Milli-Q water, confirming the presence of cationic groups (Table 1). Additionally, the terpolymers (LPs and CPs) were assessed in PBS (pH 7.4) (Table 1 and ESI, Table S2†), where a significant decrease in both *D*_h_ and *ζ* was observed compared to the terpolymers analyzed in Milli-Q water (Table 1 and ESI, Table S2†), in agreement with our previous antibacterial study.^37^

### Antibacterial activity

To assess the influence of topology, hydrophobic monomer type, and monomer composition on the antibacterial activity of our terpolymers, we determined the minimum inhibitory concentrations (MIC_90_). MIC_90_ is defined as the lowest concentration of a compound required to inhibit 90% of bacterial growth by compared to untreated controls after a 20 h incubation period. We used colistin, gentamicin, and ciprofloxacin, as reference antibacterial agents (control). Four Gram-negative strains, including *Escherichia coli* (EC K12), *Pseudomonas aeruginosa* (PA, ATCC 27853; and PA37, a multidrug-resistant (MDR) strain), *Acinetobacter baumannii* (AB, ATCC 19606), and a Gram-positive strain *Staphylococcus aureus* (SA, ATCC 29213) were selected to screen the activity of our LPs and CPs.

Among terpolymers containing (E) hydrophobic monomer (LPEs: LPE-20 and LPE-30; CPEs: CPE-20 and CPE-30), LPE-20 displayed a MIC_90_ of 32 μg mL^−1^ against *E. coli* (EC K12) and 64–128 μg mL^−1^ against *P. aeruginosa* (PA 27853), while LPE-30 showed enhanced activity with MIC_90_ of 8 μg mL^−1^ (EC K12) and 32 μg mL^−1^ (PA 27853) (Table 2). For the cyclic counterparts, CPE-20 displayed a slightly higher MIC_90_ values of 64 μg mL^−1^ (EC K12) and 256 μg mL^−1^ (PA 27853), whereas CPE-30 exhibited improved potency with MIC_90_ of 16–32 μg mL^−1^ (EC K12) and 128 μg mL^−1^ (PA 27853) (Table 2).

**Table 2:** Antibacterial activity of synthetic terpolymers and antibiotics^a^

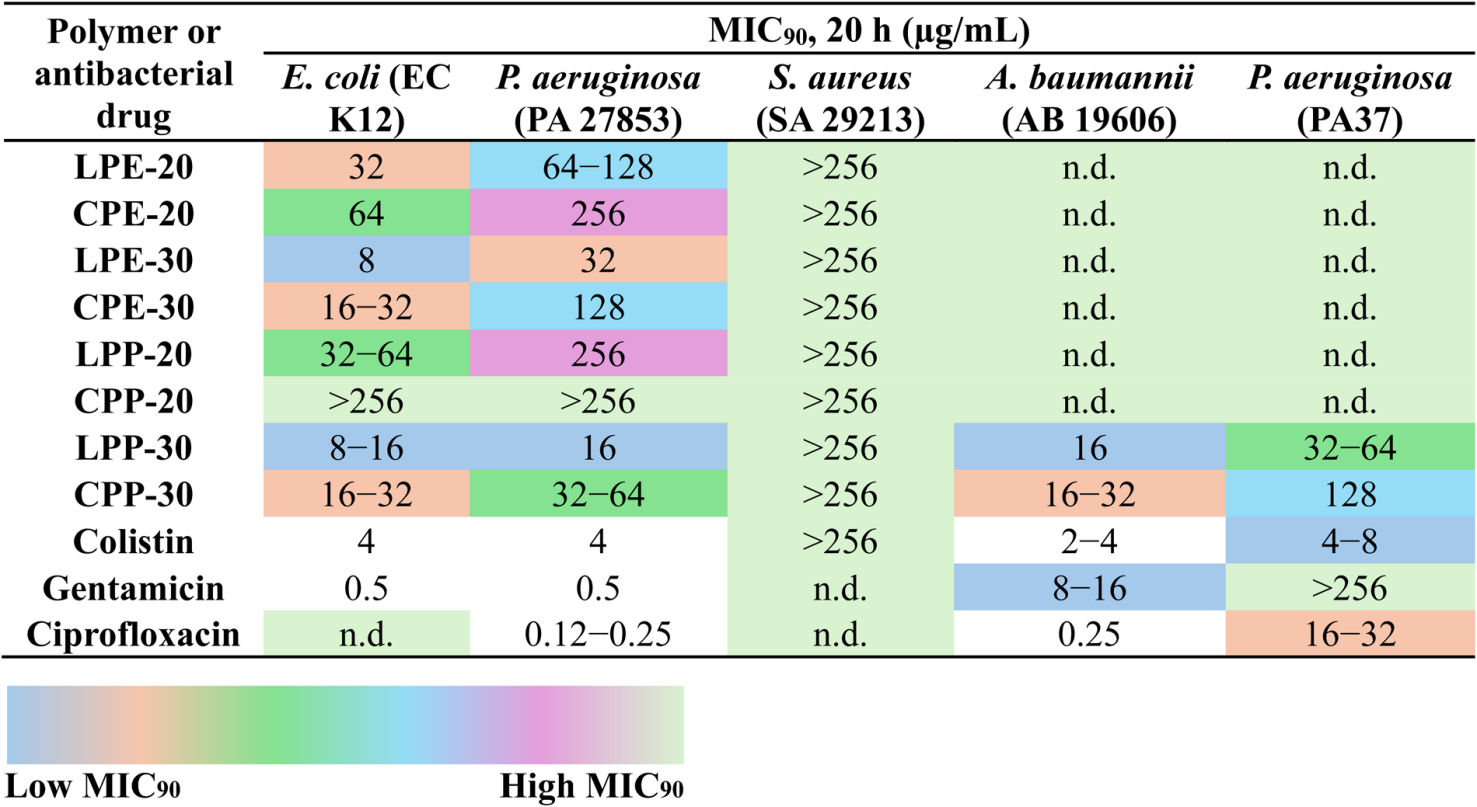

a Notes: The antibacterial activity of the terpolymers was evaluated by determining the minimum inhibitory concentration (MIC_90_), defined as the lowest concentration required to inhibit 90% of bacterial growth compared to untreated controls after 20 hours of incubation. MIC_90_ values, expressed in μg mL^−1^ of the terpolymers, or antibiotics (colistin, gentamicin, and ciprofloxacin) tested against *E. coli* (EC K12), *P. aeruginosa* (PA 27853), *S. aureus* (SA 29213), *A. baumannii* (AB 19606), and *P. aeruginosa* (PA37, MDR strain). The results are presented in Table 2, with a color scale indicating relative antibacterial efficacy: blue for low MIC_90_ values (high efficacy) and green for high MIC_90_ values (low efficacy). All experiments were performed in triplicate (*n* = 3).

Among the terpolymers containing (P) as the hydrophobic monomer, LPP-30 emerged as the most potent, exhibiting MIC_90_ values of 8–16 μg mL^−1^ against *E. coli* (EC K12) and 16 μg mL^−1^ against *P. aeruginosa* (PA 27853). This was a significant improvement over LPP-20, which showed MIC_90_ values of 32–64 μg mL^−1^ (EC K12) and 256 μg mL^−1^ (PA 27853) (Table 2). Interestingly, CPP-20 was ineffective against both EC K12 and PA 27853 even at the highest tested concentration (256 μg mL^−1^), while CPP-30 demonstrated a significant improvement in antibacterial activity, with MIC_90_ values of 16–32 μg mL^−1^ (EC K12) and 32–64 μg mL^−1^ (PA 27853) (Table 2). To further confirm these findings, a 3D tomographic microscope was used to visualize a 96-well microplate containing EC K12 treated at MIC_90_ concentrations with LPP-30 and CPP-30. This analysis revealed minimal bacterial growth compared to the untreated control (without polymer), validating the MIC_90_ values obtained (ESI, Fig. S21†). Overall, these results highlight that varying the hydrophobic monomer ratio (20–30%) within the same hydrophobic group can influence MIC_90_ values up to 2–4-fold. Furthermore, terpolymers containing the (P) hydrophobic group generally displayed slightly higher antibacterial activity against *P. aeruginosa* compared to those with the (E) group, with LPP-30 being the most potent overall. Importantly, LPs consistently outperformed their cyclic counterparts against the tested Gram-negative bacteria (Table 2).

Further investigation of antibacterial activity (MIC_90_) against Gram-positive bacterium (SA 29213) revealed that all our terpolymers, both LPs and CPs, were inactive even at the highest concentration (256 μg mL^−1^) (Table 2). This finding is consistent with our previous studies using similar type of monomer and composition.^11,73,74^ The observed difference in antibacterial activity of the terpolymers against Gram-negative and Gram-positive bacteria can be attributed to their distinctive cell wall structures. Gram-negative bacteria possess a thin, loosely cross-linked peptidoglycan layer covered by an outer membrane with lipopolysaccharides, providing potential anchoring sites for the cationic groups of our polymers.^75^ In contrast, Gram-positive cell walls have multiple layers of densely cross-linked peptidoglycan, creating a “sieving effect” that hinders the penetration of bulky hydrophobic molecules.^76,77^ The absence of an outer lipopolysaccharide layer, combined with this sieving effect, likely contributes to the inactivity of our terpolymer's against Gram-positive bacteria.^78^ Furthermore, Table 2 shows the MIC_90_ values for the control antibiotics, colistin and gentamicin exhibited values of 4 μg mL^−1^ and 0.5 μg mL^−1^, respectively against *E. coli* (EC K12), while ciprofloxacin demonstrated a range of 0.12 to 0.25 μg mL^−1^ against PA 27853.

Despite our terpolymers exhibiting lower antimicrobial activity compared to conventional antibiotics against standard strains, we expanded our investigation to include two clinically relevant bacterial strains: *A. baumannii* (AB 19606), identified by the WHO as a critical priority pathogen,^6,79^ and a multidrug-resistant (MDR) strain of *P. aeruginosa* (PA37). This allowed us to assess the potential of our terpolymers against challenging infections where traditional antibiotics may be less effective or ineffective. We selected our most potent terpolymers, LPP-30 and CPP-30, for further testing against these additional Gram-negative bacteria. Pleasingly, both terpolymers demonstrated good antibacterial activity against AB 19606 and PA37. Specifically, LPP-30 showed MIC_90_ values of 16 μg mL^−1^ and 32–64 μg mL^−1^ against AB 19606 and PA37, respectively, while CPP-30 exhibited MIC_90_ values of 16–32 μg mL^−1^ and 128 μg mL^−1^ against these same strains (Table 2). As a positive control, commercial antibiotic agents were tested, demonstrating expected MIC_90_ values against PA37 and AB 19606 (Table 2). Notably, against MDR strain (PA37), gentamicin was ineffective even at the highest tested concentration (256 μg mL^−1^), while ciprofloxacin showed significantly reduced efficacy with an MIC_90_ of 16–32 μg mL^−1^, which is 128-fold increase compared to the PA 27853 (0.12–0.25 μg mL^−1^). Colistin retained some activity against PA37, but with a lightly higher MIC_90_ value of 4–8 μg mL^−1^.^80^ Overall, PA37 demonstrated slightly lower sensitivity compared to EC K12, PA 27853, and AB 19606. However, the relatively low MIC_90_ values and the smaller fold change in MIC_90_ against PA37, compared to the significant loss of efficacy observed with gentamicin and ciprofloxacin, highlight the promising potential of our terpolymers, particularly LPP-30 and CPP-30, to combat MDR Gram-negative bacterial infections.^73,81^

**Fig. 3:**
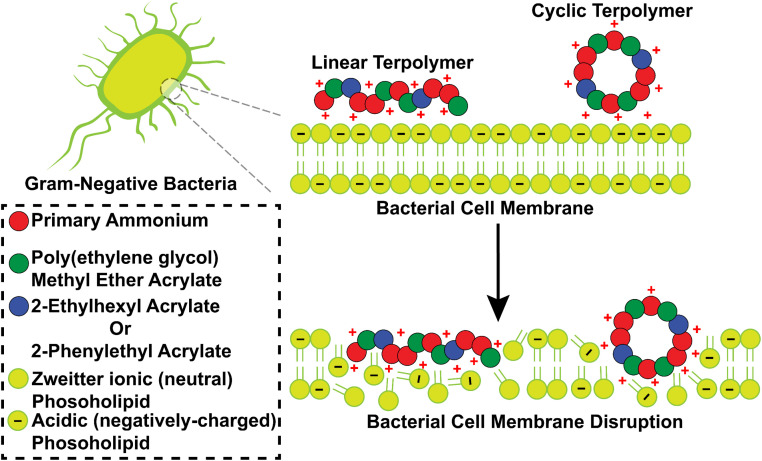
Proposed mechanism of interaction of cationic linear polymers (LPs) and cyclic polymers (CPs) with Gram-negative bacterial cell membranes. (A) Due to their high flexibility and mobility, LPs can maximize contact with the bacterial membrane, promoting stronger electrostatic interactions and efficient membrane disruption. (B) Conversely, the constrained conformation of CPs limits their surface contact and interaction with the bacterial membrane, potentially reducing their antibacterial activity.

In all tested different bacterial strains, our LPs exhibited superior antibacterial efficacy compared to their CPs, despite having nearly similar zeta potentials in Milli-Q water (Table 1). Additionally, we observed self-assembly of the terpolymers which may be due to the high concentrations used during DLS analysis (Table 1). However, it is important to note that these assemblies may not form at the lower antibacterial study concentration (Table 2) or disassemble near the bacterial membrane. We attribute this difference in efficacy to the inherent structural properties of LPs. The linear, flexible chains of LPs offer greater conformational freedom, facilitating enhanced mobility and potentially faster diffusion through bacterial membranes compared to the more constrained CPs. Furthermore, the flexibility of LPs allows them to readily adapt upon contact with the bacterial surface, maximizing interactions between their cationic groups and the negatively charged bacterial membrane. This enhanced electrostatic attraction, coupled with hydrophobic interactions between the polymer backbone and the phospholipid bilayer, promotes more efficient membrane disruption and ultimately, bacterial cell death (Fig. 3). Conversely, the closed-loop structure of CPs results in more compact structure as noted by DLS in comparison to LPs (Table 1), which limits their flexibility, potentially hindering their interaction with the bacterial membrane (Fig. 3). This reduced molecular mobility could weaken their binding affinity and limit their ability to disrupt the membrane effectively, which is in agreement with a previous study on cyclic glycopolymers.^72^

### Cytoplasmic membrane potential study

**Fig. 4:**
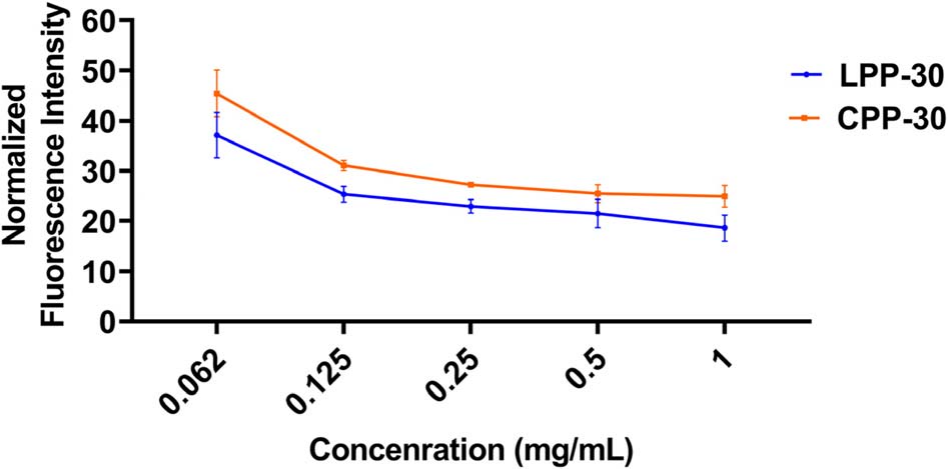
The red fluorescence (emitting at 670 nm) intensity of Gram-negative, *E. coli* in the presence of different concentrations of LPP-30 and CPP-30 terpolymers.

To validate our hypothesis that CPs and LPs interact differently with bacterial membranes, we conducted a cytoplasmic bacterial membrane (CBM) permeabilization study. Previous studies, including our own, have established that cationic amphiphilic polymers exert their antibacterial effects through membrane disruption *via* electrostatic and hydrophobic interactions.^82–84^ Focusing our most potent terpolymers, LPP-30 and CPP-30, we measured the membrane potential of Gram-negative *E. coli* (EC K12) bacteria treated with these polymers. We employed carbocyanine dye 3,3′-diethyloxacarbocyanine iodide, which exhibits red fluorescence when accumulated in bacterial cytosol indicative of intact CBM, but shifts to green fluorescence upon membrane depolarization.^38^ By monitoring the red fluorescence intensity (emission at 670 nm), we assessed the extent of membrane disruption. As shown in Fig. 4, LPP-30 induced a greater decrease in red fluorescence intensity compared to CPP-30 terpolymer across a concentration range of 0.0625–1 mg mL^−1^ in a dose-dependent manner after a 5 min incubation. At the lowest concentration tested (0.0625 mg mL^−1^), the normalized fluorescence intensities of LPP-30 and CPP-30 were approximately 45% and 37%, respectively. This indicates that LPP-30 causes a slightly greater reduction in CBM depolarization than CPP-30, supporting a marginally superior membrane disruption capability for LP.

### Bacteria killing study

**Fig. 5:**
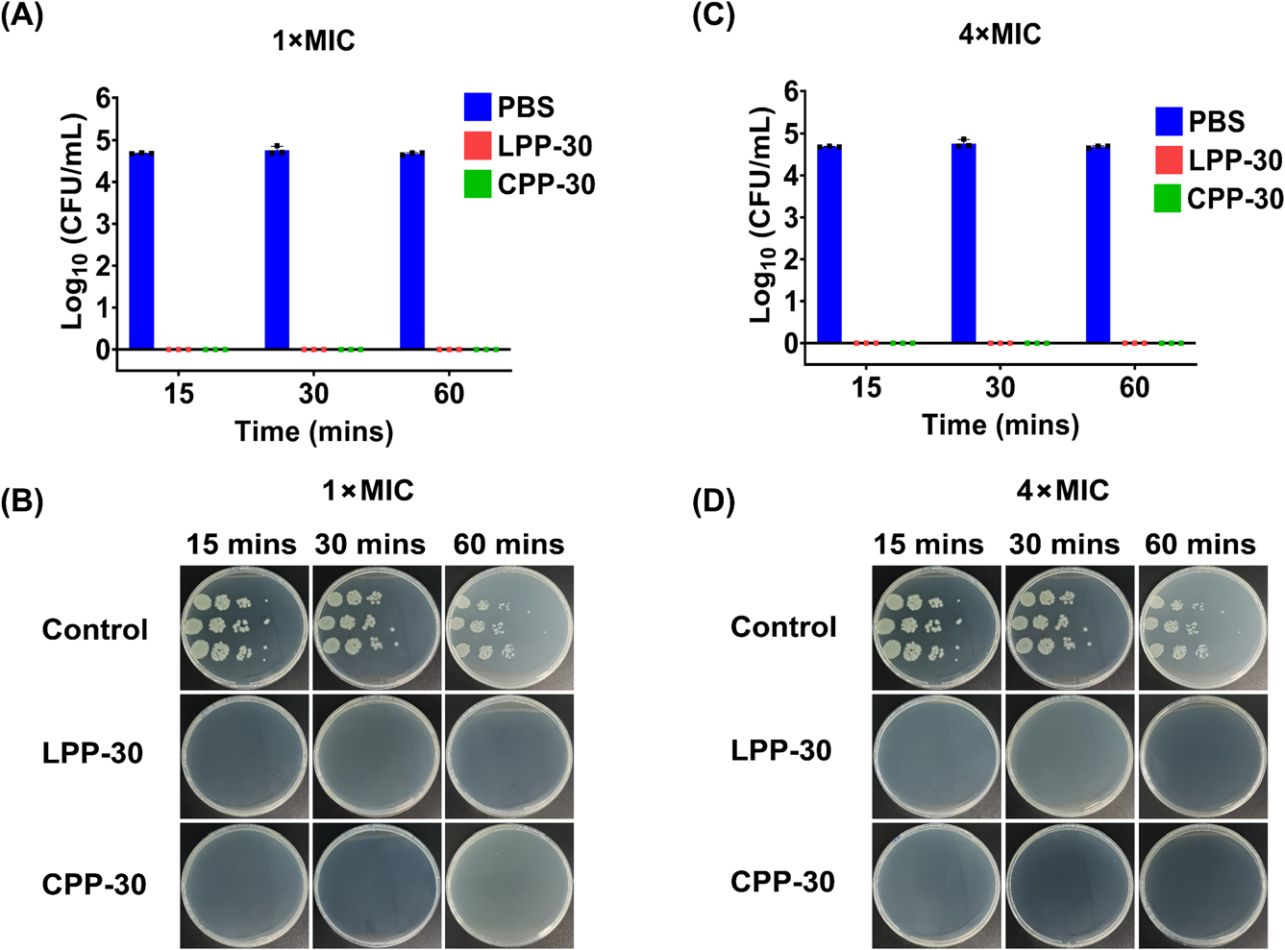
Time-kill kinetics of LPP-30 and CPP-30 against *P. aeruginosa* PA 27853 cells, determined *via* CFU assay, using an initial concentration of PA 27853 cells of 4.7 × 10^5^ CFU mL^−1^. The total PA 27853 bacterial cells killed after incubation with different concentration of the terpolymers at 37 °C in PBS (pH 7.4) was plotted against time. Figures (A and B) and (C and D) display the time-kill curves for PA 27853 cells treated with LPP-30 and CPP-30 terpolymers, respectively, at 1×MIC and 4×MIC concentrations for 15, 30, and 60 minutes (*n* = 3). The corresponding MIC_90_ values for LPP-30 and CPP-30 against PA 27853 are provided in Table 2.

The bactericidal efficacy of LPP-30 and CPP-30 was further investigated against PA 27853 using a colony forming unit (CFU) assay at different timepoints (15, 30, and 60 minutes) of treatment in a PBS (pH 7.4). Impressively, both terpolymers, at concentrations of 1×MIC (Fig. 5A and B) and 4×MIC (Fig. 5C and D), completely eradicated (99.99%) PA 27853 bacterial cells within the first 15 minutes, resulting in no detectable CFUs compared to the untreated control sample (4.7 × 10^5^ CFU mL^−1^). Extending the treatment durations to 30 and 60 minutes resulted in complete bacterial killing, highlighting the rapid bactericidal kinetics and potent activity of our terpolymers against PA 27853 (Fig. 5). This rapid bactericidal effect is consistent with previous findings using similar amphiphilic methacrylate copolymers against *E. coli*, where nearly 99.9% of cells were killed within 15–60 minutes at 2×MIC concentrations.^85^

### Hemolysis and cytotoxicity assay

Given the known cytotoxicity of antimicrobial peptides towards mammalian cells,^23^ it is crucial to assess the biocompatibility of our terpolymers. To assess this, we determined the hemolytic activity (HC_50_) of all terpolymers (LPs and CPs) using defibrinated sheep red blood cells (sRBCs). HC_50_ is defined as the minimum concentration required to induce 50% hemolysis of sRBCs.

Among the terpolymers containing the (E) hydrophobic group, the LPEs (LPE-20 and LPE-30) and CPEs (CPE-20 and CPE-30) displayed distinct hemolytic profiles (Table 3). LPE-20 exhibited HC_50_ values exceeding 1000 μg mL^−1^, whereas CPE-20 remarkably showed no hemolysis even at the highest tested concentration of 2000 μg mL^−1^. Increasing the hydrophobic monomer ratio to 30% significantly reduced the HC_50_ values for both LPE-30 and CPE-30 to below 125 μg mL^−1^ (Table 3). For terpolymers containing the (P) hydrophobic group (LPPs and CPPs), LPP-20 showed HC_50_ values above 2000 μg mL^−1^, a two-fold increase compared to LPE-20 (>1000 μg mL^−1^) (Table 3). Similar to CPE-20, CPP-20 also showed no hemolytic activity. However, increasing the hydrophobic monomer content to 30% led to a stark contrast in hemolytic behavior. Indeed, LPP-30 was highly hemolytic with HC_50_ below 125 μg mL^−1^, while CPP-30 maintained excellent hemocompatibility (>2000 μg mL^−1^), representing a 16-fold improvement over LPP-30 (Table 3). These results demonstrates that while increasing hydrophobicity significantly reduced the HC_50_ values of LP, consistent with previous reports,^11,30,38,78,86^ the impact on CPs was less pronounced. This suggests that the cyclic topology, with its reduced flexibility and chain mobility, may confer a degree of protection against hemolysis, even at higher hydrophobic monomer ratios. As APs with complex topologies have been reported to cause hemagglutination,^40,41,47^ we decided to conduct a hemagglutination assay, utilizing a previously published protocol.^40^ Our results evidenced no hemagglutination at concentrations up to 2000 μg mL^−1^ for all our polymers. Moreover, our control antibiotic, colistin, displayed no hemolytic activity at concentrations up to 2000 μg mL^−1^. To further assess biocompatibility, we evaluated the cytotoxicity of our terpolymers against the RAW 264.7 cell line using a standard CCK-8 cell viability assay.^83^ We determined the minimum concentration required to inhibit cell viability by 50% (IC_50_, Table 3). Consistent with the hemolysis results, our IC_50_ measurements revealed that CPs (IC_50_ values >64 to >256 μg mL^−1^) were generally more biocompatible than LPs (IC_50_ values >32 to >128 μg mL^−1^) (Table 3). This trend aligns with recent findings on the enhanced biocompatibility of cationic cyclic copolymers.^48,50^ Furthermore, increasing the hydrophobic content from 20% to 30% in both LPs and CPs led to lower IC_50_ values, consistent with the trend observed in the hemolysis study (Table 3). This suggests that increased hydrophobicity is associated with increased cytotoxicity, regardless of topology. Interestingly, only minor differences in the IC_50_ values between the (E) and (P) hydrophobic monomer groups, indicating that the specific type of hydrophobic monomer had a relatively minor impact on cytotoxicity compared to the overall hydrophobicity level. Lastly, we also assessed the cytotoxicity of common antibiotics: colistin (>128 μg mL^−1^), gentamicin (>512 μg mL^−1^), and ciprofloxacin (>32 μg mL^−1^) (Table 3). The obtained cytotoxicity of antibiotics were comparable to other reported studies.^38,87,88^

**Table 3:** Summary of hemolytic activity (HC_50_), cytotoxicity (IC_50_), PA 27853 (MIC_90_), and therapeutic index (TI) values of antibacterial terpolymers and antibiotics^a^

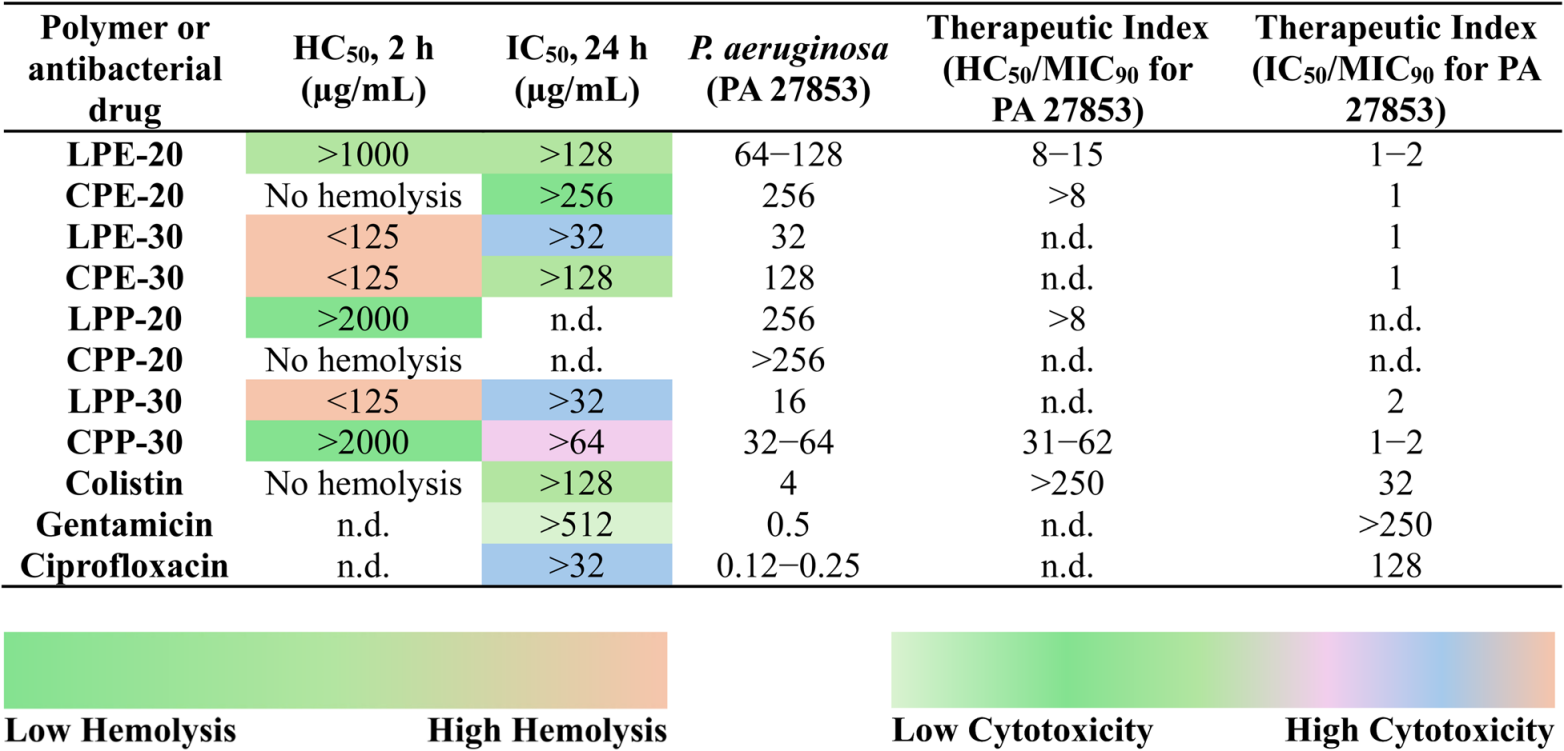

a Notes: (1) The scale shows HC_50_ and IC_50_ values. HC_50_ and IC_50_ values were expressed in μg mL^−1^ of the antibacterial terpolymers and antibiotics. HC_50_ represents the minimum terpolymer concentration causing 50% hemolysis of sheep red blood cells (sRBCs), IC_50_ is defined as the minimum concentration inhibiting 50% of cell viability. The therapeutic index (TI) was calculated by taking the ratio of HC_50_ or IC_50_ and MIC_90_ for *P. aeruginosa* (PA 27853). n.d. not determined. All experiments were conducted in triplicate (*n* = 3). (2) Hemolysis was assessed using a maximum concentration of 2000 μg mL^−1^. “No hemolysis” indicates no RBC lysis at this concentration. While some hemolysis may have been detected at concentrations exceeding 2000 μg mL^−1^, the HC_50_ (concentration causing 50% hemolysis) was consistently above this threshold. For therapeutic index (TI) calculations (HC_50_/MIC_90_ for PA 27853), we conservatively used >2000 μg mL^−1^ as the HC_50_ in all cases.

To evaluate the selectivity of our terpolymers and antibiotics, we calculated their therapeutic index (TI) values, a standard measure of an antibacterial agent's preferential targeting of bacterial cells over mammalian cells.^23^ TI values were determined by dividing the HC_50_ or IC_50_ by the MIC_90_ values against PA 27853 (*i.e.*, TI = HC_50_/MIC_90_ or IC_50_/MIC_90_) (Table 3) according to previous published methodologies.^37,71^ As shown in Table 3, colistin exhibited a TI value (HC_50_/MIC_90_) greater than 250, while the IC_50_/MIC_90_ values for colistin (32), gentamicin (>250), and ciprofloxacin (128) were higher compared to those of our antibacterial terpolymers (Table 3). Similar TI values for antibiotics have been reported in different antimicrobial polymer studies.^37,41,89^ Interestingly the TI (IC_50_/MIC_90_) values of both LPs and CPs were comparable, ranging from 1 to 2 (Table 3). However, the calculated TI (HC_50_/MIC_90_) value for CP was notably higher than those for LPs. Specifically, CPP-30 showed TI values 31–62, whereas LPE-20 and LPP-20 had TI values of 8–15 and >8, respectively (Table 3).

Taken together, these results highlight the superior biocompatibility and selectivity of CPs compared to LPs. We attribute this difference to the constrained and less flexible nature of CPs, which likely reduces their non-specific interactions with mammalian cell membranes and sRBCs, leading to minimal to no toxicity. Our findings underscore the significant impact of polymer topology and the optimization of hydrophilic/hydrophobic balance in minimizing cytotoxicity and hemolysis and improving TI.

## Conclusions

In this study, we successfully synthesized a library of amphiphilic cationic statistical terpolymers with a linear and cyclic topology, designed to mimic the antimicrobial properties of HDPs. The LPs were prepared by RAFT polymerization and subsequently cyclized into CPs *via* a simple and efficient light-induced Diels–Alder click reaction. We systematically investigated the antibacterial properties and biocompatibility of these LPs and CPs, varying the hydrophobic monomer types (E and P) and their content (20–30%). Our results demonstrated that LPs consistently exhibited superior antibacterial activity (MIC_90_) against 4 Gram-negative bacterial strains compared to their cyclic counterparts. Increasing the hydrophobic content (from 20% to 30%) enhanced their antibacterial activity for both LPs and CPs, and terpolymers containing the (P) hydrophobic group showed slightly superior performance compared to those with the (E) group. Notably, our lead terpolymers, LPP-30 and CPP-30, displayed promising inhibitory effects against the MDR Gram-negative strain *P. aeruginosa* (PA37), outperforming the antibiotics gentamicin and ciprofloxacin. Furthermore, preliminary mechanistic studies revealed that both LPP-30 and CPP-30 rapidly disrupted bacterial cell membranes, resulting in complete killing of *P. aeruginosa* within 15 minutes. Importantly, CPs exhibited minimal to no hemolytic activity, showcasing a significantly improved safety profile compared to their linear counterparts.

This proof-of-concept study highlights the advantage of tuning polymer topology and optimizing hydrophilic/hydrophobic balance to address the cytotoxicity and selectivity issues associated with HDPs. By systematically exploring the structure–activity relationships of linear *versus* cyclic terpolymers with varying compositions and ratios of hydrophobic monomers, we have provided valuable insights for the rational design of effective and safe APs. These findings pave the way for the development of novel therapeutic strategies to combat the growing threat of antimicrobial resistance.

## Author contributions

Conceptualization, methodology, investigation, formal analysis, and writing – original draft: Md.A., methodology, investigation, and validation: W. Y., L. Y., V. K. K., resources, methodology, investigation, and validation: Y. Z., P. L., A. W., C. F., conceptualization, resources, writing – review & editing, and supervision: C. B.

## Conflicts of interest

There are no conflicts to declare.

## Supplementary Material

SC-015-D4SC05797J-s001

## Data Availability

All supporting data is provided in the ESI.†
