# Supplementary material for: Effect of cyclic topology versus linear terpolymers on antibacterial activity and biocompatibility: antimicrobial peptide avatars
Source: Chem Sci. 2024 Oct 24;15(45):19057–69. doi: 10.1039/d4sc05797j (PMC11520352; doi:10.1039/d4sc05797j)
Supplement: SC-015-D4SC05797J-s001 [file SC-015-D4SC05797J-s001.pdf]

Supplementary Information (SI) for Chemical Science.
This journal is © The Royal Society of Chemistry 2024

Supporting Information

Effect of cyclic topology versus linear terpolymers on antibacterial activity and biocompatibility: Antimicrobial peptide avatars

Md Aquib,[a] Wenting Yang,[b] Luofeng Yu,[a,c] Vinod Kumar Kannaujiya,[a] Yuhao Zhang,[b] Peng Li,[c] Andrew Whittaker,[b] Changkui Fu,*[b] Cyrille Boyer*[a]

[a] *Cluster for Advanced Macromolecular Design (CAMD) and Australian Centre for NanoMedicine (ACN), School of Chemical Engineering, UNSW Australia, Sydney, NSW 2052, Australia*

[b] *Australian Institute for Bioengineering and Nanotechnology, The University of Queensland, St Lucia, Queensland, 4072, Australia*

[c] *Frontiers Science Center for Flexible Electronics (FSCFE), Xi'an Institute of Flexible Electronics (IFE) and Xi'an Institute of Biomedical Materials & Engineering (IBME), Northwestern Polytechnical University, 127 West Youyi Road, Xi'an 710072, China*

Email: cboyer@unsw.edu.au; changkui.fu@uq.edu.au

Experimental section

Materials

2-Ethylhexyl acrylate (Sigma-Aldrich, 98%), *tert*-butyl (2-acrylamidoethyl) carbamate (Boc-AEAm; Ambeed, 95%), 2-phenylethyl acrylate (Tokyo Chemical Industry, > 98%), poly(ethylene glycol) methyl ether acrylate (Sigma-Aldrich, average M_n = 480 g/mol), trifluoroacetic acid (TFA; Sigma-Aldrich, 99%), Triton™ X-100 (Sigma-Aldrich), deuterated dimethyl sulfoxide (DMSO) (Cambridge Isotope Laboratories, Inc), deuterated chloroform (Cambridge Isotope Laboratories, Inc), high purity liquid chromatography (HPLC) grade dimethylacetamide (DMAc, Sigma-Aldrich), dichloromethane (DCM; Merck), were used as received. Acetone, *n*-hexane, magnesium sulfate ($MgSO_4$), diethyl ether, and DMSO, were obtained from Chem-Supply and used as received. Deionized (DI) water was acquired by a Milli-Q water purification system with a resistivity of 18.2 mΩ/cm. Defibrinated sheep red blood cells (sRBCs) were purchased from Serum Australis (Australia). 2,2′-azobis(2-methylpropionitrile) (AIBN; Sigma-Aldrich, 0.2 M in toluene), Colistin sodium methanesulfonate (Sigma-Aldrich), Ciprofloxacin (Sigma-Aldrich, ≥ 98%), Gentamicin sulphate (Enzo Life Sciences), Komodo compact reptile lamp (UVB 10% ES, 15 W) was purchased from Petbarn (Australia). 3,3′-diethyloxacarbocyanine iodide ($DiOC_2(3)$; Sigma-Aldrich, 98%), ethylenediaminetetraacetic acid (EDTA; Sigma-Aldrich), D-(+)-glucose (Sigma-Aldrich, ≥ 99.5%), Oxoid™ phosphate buffered saline (PBS) tablet (Thermo Scientific™).

Synthesis of functional RAFT agent for the preparation of terpolymers

The synthesis of functional RAFT agent for the preparation of linear and cyclic terpolymers was conducted according to the procedure reported in previous literature with slight modifications.[1]

a. Synthesis of 2-methoxy-6-methylbenzaldehyde

2,3-Dimethylanisole (2.5 g, 18.4 mmol), copper sulfate (3.0 g, 18.7 mmol), and potassium peroxodisulfate (14.9 g, 55.1 mmol) were added to a round bottom flask containing a mixture of acetonitrile and water (180 mL, 1/1). The suspension was heated at 90 °C while stirring until the starting reagents were consumed according to thin layer chromatography (TLC) analysis. The product was purified by silica chromatography

using *n*-hexane and ethyl acetate (5/1, v/v) as the eluent. Yield: 2.0 g. (**supplementary information (SI), Figure S1A**).

^{1}H NMR ($CDCl_3$, 400 MHz) δ (ppm): 10.65 (s, 1H, C*H*O), 7.38 (t, 1H, Ar*H*), 6.82 (dd, 2H, Ar*H*), 3.90 (s, 3H, OCH_3), 2.57 (s, 3H, CH_3)

MS+Na: expected 173.17; found 173.25.

b. Synthesis of 2-hydroxy-6-methylbenzaldehyde

2-methoxy-6-methylbenzaldehyde (2 g, 13.3 mmol, 1.00 eq) was dissolved in anhydrous DCM (30 mL) in a flask which was placed in an ice bath. To this solution was added $AlCl_3$ (5.34 g, 40.0 mmol, 3.00 eq). After addition the mixture was moved to room temperature and stirred for overnight. Subsequently, the reaction was quenched by dropwise addition of water. The crude product was extracted by DCM (100 mL) and dried over $MgSO_4$. The product was purified by silica gel chromatography using *n*-hexane and ethyl acetate (4:1 v/v) as the eluent. Yield: 1.2 g. (**Figure S1B**).

^{1}H NMR ($CDCl_3$, 400 MHz) δ (ppm): 11.91 (s, 1H, O*H*), 10.33 (s, 1H, C*H*O), 7.38 (t, 1H, Ar*H*), 6.76 (dd, 2H, Ar*H*), 2.61 (s, 3H, CH_3).

MS+H: expected 137.16; found 137.15.

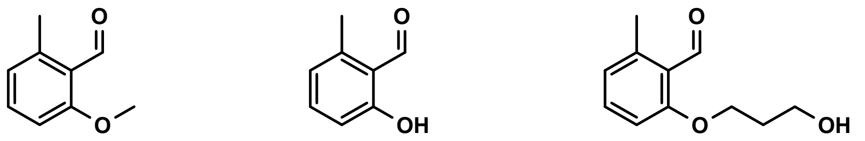

(A) **(B)** **(C)**

Figure S1. Chemical structure of synthesized (A) 2-methoxy-6-methylbenzaldehyde. (B) 2-hydroxy-6-methylbenzaldehyde. (C) 2-(3-hydroxypropoxy)-6-methylbenzaldehyde.

c. Synthesis of 2-(3-hydroxypropoxy)-6-methylbenzaldehyde

K_2CO_3 (5.66 g, 41 mmol) was added to a flask containing 50 mL anhydrous acetone. 2-hydroxy-6-methylbenzaldehyde (3.0 g, 22 mmol) was added to the flask followed by addition of 3-iodo-1-propanol (6.66

g, 35.8 mmol). The reaction was left at 50 °C for 120 h. The product was purified by silica gel chromatography using *n*-hexane and ethyl acetate (2/1, v/v) as the eluent. Yield: 2.7 g. (**Figure S1C**).

^{1}H NMR ($CDCl_3$, 400 MHz) δ (ppm): 10.60 (s, 1H, C*H*O), 7.35 (t, 1H, Ar*H*), 6.80 (dd, 2H, Ar*H*), 4.19 (t, 2H, ArOCH_2), 3.85 (t, 2H, CH_2OH), 2.53 (s, 3H, CH_3), 2.06 (m, 2H, C$H_2$$CH_2$OH).

MS+Na: expected 217.22; found 217.33.

d. Synthesis of functional RAFT agent

3-(2-formyl-3-methylphenoxy)propyl 4-cyano-4-((phenylcarbonothioyl)thio)pentanoate

4-cyano-4-(phenylcarbonothioylthio)pentanoic acid (CPADB) (1.0 g, 3.58 mmol), 2-(3-hydroxypropoxy)-6-methylbenzaldehyde (0.9 g, 4.63 mmol) (**I, Figure 1A**) and 4-dimethylaminopyridine (DMAP) (10 mg) were dissolved in 50 mL THF followed by addition of dicyclohexylcarbodiimide (DCC) (1.1 g, 5.33 mmol) (**Figure 1A**). The reaction was left at room temperature while stirring for 24 h. The product was purified by silica gel chromatography using *n*-hexane and ethyl acetate (3/1, v/v) as the eluent. Yield: 1.22 g.

^{1}H NMR (DMSO-d6, 400 MHz) δ (ppm):10.55 (s, 1H, C*H*O), 7.91 (d, 2H, Ar*H*), 7.69 (t, 1H, Ar*H*), 7.49 (t, 3H, Ar*H*), 7.05 (d, 1H, Ar*H*), 6.86 (d, 1H, Ar*H*), 4.25 (t, 2H, ArOCH_2), 4.18 (t, 2H, COOCH_2), 2.63 (m, 2H, C$H_2$$CH_2$COO), 2.46 (s, 3H, ArC$H_3$), 2.11 (m, 4H, C$H_2COOCH_2CH_2$), 1.91 (s, 3H, C(CN)C$H_3$) (**Figure S2**).

MS+Na: expected 478.58; found 478.39.

Synthesis of linear terpolymers

The amphiphilic cationic statical linear terpolymers (LPs) were synthesized using established protocol with minor modifications.[2] In a typical polymerization, stock solutions of each monomer (Boc-AEAm, 2-ethylhexyl acrylate/2-phenylethyl acrylate, and poly(ethylene glycol) methyl ether acrylate) were prepared at a concentration of 33% (w/w) in DMSO. RAFT agent was added to a 4 mL glass vial in an amount corresponding to the targeted X_n of 50. Subsequently, based on the target polymer composition, the required amounts of the prepared monomer stock solutions were combined in the vial containing RAFT agent, and additional DMSO was added to achieve a final monomer concentration of 10% (w/w). A radical initiator (AIBN) was added at a concentration of 0.4 eq. to the RAFT agent. The glass vial was tightly sealed with a

rubber septum, and the headspace was degassed with N_2 for 20 mins. Following this, the vial was exposed to 70 °C and stirred for 30 h. After polymerization, the final polymer solution was analyzed by proton nuclear magnetic resonance (^{1}H NMR) spectroscopy and size-exclusion chromatography (SEC) to evaluate the monomer conversion, polymer composition, and molecular weight distribution (MWD). The resulting polymers were purified by precipitating into ice-chilled *n*-hexane: diethyl ether mixture (7:3 v:v, 12 mL). The precipitate was isolated by centrifugation (9000 rpm for 3 mins), dissolved in acetone, and precipitated twice more. The Boc-protected terpolymers were dried in vacuo before removing the Boc-group (**Figure S4**).

Synthesis of cyclic terpolymer

All of the amphiphilic cationic statical cyclic terpolymers (CPs) were prepared by the same procedure for ring-closing the linear precursors,[3] with minor modifications. Briefly, 25 mg of Boc-protected LP was dissolved in 50 mL of DCM (0.5 mg/mL) and added to a syringe pump. The flow rate of the syringe pump was set to 6.25 mL/h and maintained for 8 h, allowing the solution to drop into a round-bottom flask containing 950 mL of DCM. This flask was subjected to UVB light irradiation and continuous stirring. The reaction mixture was then further stirred and irradiated for an additional 4 h. After completion of the reaction, the CPs were conveniently collected by evaporating the DCM. Subsequently, the terpolymer solution was analyzed using ^{1}H NMR spectroscopy and SEC to evaluate the cyclization and the MWD. The resulting terpolymers were purified by precipitating them into an ice-chilled mixture of *n*-hexane and diethyl ether (7:3 v/v, 12 mL). The precipitate was collected by centrifugation at 9000 rpm for 3 mins, dissolved in acetone, and subjected to two additional precipitation steps. Finally, the Boc-protected terpolymers were dried under vacuum before the Boc groups were removed (**Figure S4**).

Deprotection of terpolymers

In accordance with our group's established procedure,[4] TFA was used to eliminate Boc-protecting groups. Specifically, the terpolymer (LP and CP) were dissolved in DCM (~7% (w/w) terpolymer). 20 mol of TFA to Boc groups was added. After stirring for 4 h at ambient temperature, the mixture was precipitated into 10 mL ice-chilled diethyl ether. Centrifugation (9000 rpm for 3 mins) was used to separate the precipitate, which was dissolved in acetone and further precipitated twice more. After drying the terpolymer in vacuo, ^{1}H NMR analysis was utilized to determine the Boc-protective group removal (**Figure S4**).

^{1}H NMR spectroscopy

Bruker AVANCE III 400 (400 MHz, 5 mm BBFO probe) spectrometer was used to acquire ^{1}H NMR spectra. Deuterated chloroform or DMSO was utilized as a reference solvent to determine the formation of the RAFT agent, polymer conversion and composition. All experiments were carried out with a gas flow across the probes 535 L/h with sample spinning and at a temperature of 25 °C. The composition of polymers was determined employing the equation mentioned in the **SI.**

Ultraviolet–visible (UV–vis) spectroscopy

The UV–vis spectra of the synthesized RAFT agent, as well as the LPs and CPs, were recorded in DMSO solvent using a Varian Cary 300 spectrophotometer. The scans were conducted over a wavelength range of 400 to 260 nm, with a scanning rate of 600 nm/min. All measurements were performed at room temperature and were baseline-corrected using the absorbance of the DMSO solvent. For the measurements, 3 mL aliquots of the samples were placed in a 1 cm path length quartz cuvette.

Size-exclusion chromatography

A Shimadzu liquid chromatography system with a Shimadzu refractive index detector and three MIX C columns running at 50 °C was utilized for the SEC study. DMAc (containing 0.03% (w/v) LiBr and 0.05% (w/v) 2,6-dibutyl-4-methylphenol) was used as the eluent at a flow rate of 1 mL/min. Poly(methyl methacrylate) standards with molecular weights ranging from 200 to 10^6 g/mol were used to calibrate the system.

Characterization of terpolymers in aqueous media

Dynamic light scattering (DLS) and zeta-potential measurements were carried out using a Malvern Zetasizer Nano ZS apparatus, equipped with a He–Ne laser operated at $\lambda = 633$ nm and a scattering angle of 173°. All terpolymer samples were prepared in Milli-Q water and PBS (pH 7.4) at a concentration of 1.5 mg/mL and filtered using a 0.45 μm syringe filter.

Minimum inhibitory concentration

The minimum inhibitory concentration (MIC) of the various synthesized terpolymers, colistin, gentamicin, and ciprofloxacin (antibacterial drugs used as a control) were determined by the broth microdilution technique as outlined in previous studies.[2,5] The bacterial strains examined included four gram-negative strains including wild-type *Escherichia coli* strain (*E. coli*, EC K12), *Pseudomonas aeruginosa* strain PA (ATCC 27853), and multidrug-resistant strain PA37 (kindly provided by School of Optometry and Vision the University of New South Wales)[6,7], *Acinetobacter baumannii* strain AB (ATCC 19606), and a gram-positive bacterium, *Staphylococcus aureus* strain SA (ATCC 29213). Briefly, bacterial culture was grown overnight from a single colony in 10 mL of Mueller–Hinton broth (MHB) at 37 °C with continuous shaking at 180 rpm. Following this, a subculture was prepared from the overnight culture by diluting 100 μL in 10 mL of MHB, grown to mid-log phase (about 2.5 h), and then diluted to the appropriate concentration for the MIC test. A 2-fold dilution series of 100 μL of polymer and drug samples in MHB solution were added to a 96-well microplate, followed by the addition of 100 μL of the subculture suspension. The final concentration of bacteria in each well was ~5×10^5 cells/mL. Positive controls (without polymer) and negative controls (without bacteria or polymer) were also included in all the tests. The plates were then incubated at 37 °C for 20 h, and the absorbance at 595 nm was measured with a microtiter plate reader (FLUOstar Omega, BMG Labtech). MIC_{90} was defined as the lowest concentration of the sample that inhibited cell growth by at least 90% with respect to untreated control. All assays were done in two replicates and were independently repeated on three separate occasions.

Bacterial live cell imaging

To visualize the effect of the terpolymer on gram-negative *E. coli* strain EC K12, 3 μL samples were taken from the 96-well microplate containing the MIC_{90} concentration and from the positive control (without polymer). These samples were placed on 35 mm tissue culture dishes (FluoroDish, World Precision Instruments Inc., Sarasota, FL, USA). The dishes were then analyzed using a 3D tomographic microscope (3D Cell Explorer, NanoLive, Lausanne, Switzerland) equipped with digital staining software.

Membrane potential measurements

The membrane potential measurements were conducted as per previously reported method.[8,9] Briefly, the assay buffer contains 60 mM Na_2HPO_4, 60 mM NaH_2PO_4, 130 mM NaCl, 5 mM KCl, and 0.5 mM $MgCl_2$.

NaOH was used to adjust the pH to 7.0 and supplemented with 10 mM glucose. The assay buffer was sterilized before use. The fluorophore 3,3′-diethyloxacarbocyanine iodide ($DiOC_2(3)$) was used to determine the red fluorescence intensity which indicates the membrane potential of the bacteria cells. A single colony was selected and cultured in MHB at 37 °C with shaking overnight. The subculture of a gram-negative bacteria (*E. coli*, EC K12) was prepared in fresh MHB and allowed to grow to mid-log phase. The bacterial cells were collected by centrifugation (4400 rpm, 10 mins) and resuspended in 1×PBS to an OD_{600} of 1.0. The bacteria cells were then treated with 10 mM ethylenediaminetetraacetic acid (EDTA) for 5 mins and then recentrifuged to remove EDTA. EDTA-treated bacterial cells were collected and resuspended in assay buffer to an OD_{600} of 1.0. $DiOC_2(3)$ was added to bacteria cells for a final concentration of 30 μM. Different dilution of polymers, LPP-30, and CPP-30 (0.0625−1 mg/mL) were prepared in a 96-well plate, followed by the addition of assay buffer. The red fluorescence (excitation at 485-12 nm, emission at 670-10 nm) intensity was recorded after 5 mins incubation using a microtiter plate reader (FLUOstar Omega, BMG Labtech). Positive controls (bacterial cells incubation with ethanol) and negative controls (bacterial cells incubation with PBS) were included. The experiments were performed twice independently, each consisting of two replicates.

The normalized fluorescence intensity (FI) was calculated using the following equation:

$$\% FI = \frac{FI_{sample} - FI_{positive}}{FI_{negative} - FI_{positive}} \times 100\% \qquad (1)$$

Killing kinetics assays

The bactericidal study was conducted according to established methods, with minor modifications.[10] To evaluate the bactericidal activity of the terpolymers LPP-30 and CPP-30, a time-kill assay was performed against *P. aeruginosa* PA 27853 in sterile PBS (pH 7.4). The bacterial suspension was prepared similarly to the MIC assay, using sterile PBS (pH 7.4) as the dilution medium instead of MHB. LPP-30 and CPP-30 were tested at concentrations of 1×MIC and 4×MIC, respectively. These terpolymers were incubated in equal volumes with the bacterial suspension at 37 °C for 15, 30, and 60 mins with constant shaking at 100 rpm. At each time point, 10 μL aliquots were withdrawn and serially diluted 10-fold to various concentrations. The diluted bacterial solutions (10 μL) were then plated on Luria-Bertani (LB) agar and incubated for 24 h at 37 °C. Subsequently, bacterial colonies were counted, and colony-forming unit (CFU) analysis was performed.

A bacterial suspension incubated with sterile PBS served as a negative control. All assays were repeated in at least three independent experiments.

Hemocompatibility studies

The hemolytic activity of terpolymers and antibacterial drugs were determined using fresh sheep red blood cells (sRBCs) according to the previously introduced method by our group.[11] Briefly, sRBCs were diluted 1:20 in PBS (pH 7.4), pelleted by centrifugation (1000g, 10 mins), and washed three times in PBS. The sRBCs were then resuspended to achieve a 5% (v/v) concentration in PBS. Different concentrations of terpolymers (150 μL) were prepared in sterilized tubes, followed by the addition of the diluted sRBCs suspension (150 μL). Terpolymer and antibiotic concentrations tested were 2000, 1000, 500, 250, and 125 μg/mL. PBS buffer was used as a negative control, and Triton-X 100 (1% v/v in PBS) was used as a positive hemolysis control. Tubes were incubated at 37 °C for 2 h with 150 rpm shaking. Samples were then centrifuged (1000g, 8 mins), 100 μL aliquots of supernatants were transferred into a 96-well microplate, and absorbance values were read at 485 nm using a microtiter plate reader (FLUOstar Omega, BMG Labtech). All tests were performed in triplicate. The hemolysis percentage was calculated using the following formula:

$$\% \, Hemolysis = \frac{A_{polymer} - A_{negative}}{A_{positive} - A_{negative}} \times 100\% \qquad (2)$$

Where $A_{polymer}$ is the absorbance of the polymer-treated supernatant, $A_{negative}$ is the absorbance of the negative control, and $A_{positive}$ is the absorbance of the positive control.

We also conducted the hemagglutination assay of our terpolymers using a visual analysis approach, following the previously established method.[12]

Mammalian cell cytotoxicity studies

The cytotoxicity assay was performed using murine macrophage cell line, RAW 264.7 cells, following the previously reported method.[13] Briefly, RAW 264.7 cells were cultured in RPMI 1640 medium in an incubator (37 °C, 5% CO_2). The cells were seeded onto a 96-well plate at a density of 1.0×10^5 cells per well and subsequently incubated for 24 h. Afterwards the cells were incubated with polymer/medium solutions with different concentrations (16 μg/mL, 32 μg/mL, 64 μg/mL, 128 μg/mL, 256 μg/mL, and 512 μg/mL) for 24 h.

After 24 h, the polymer solution was removed and fresh medium (90 μL) containing Cell Counting Kit 8 (CCK-8) (Abcam) (10 μL) was added and incubated for another 2 h. The light intensity of the samples at an excitation wavelength of 450 nm was measured on a Tecan Infinite 200 PRO Microplate Reader. The viability of the cells treated with PBS as a positive control was normalized to 100%. The half maximal inhibitory concentration (IC_{50}) values were defined as the lowest concentration of the sample that inhibited cell viability by at least 50% compared to the untreated control. These values were analyzed using GraphPad Prism 10, employing non-linear sigmoidal curve fitting with the normalized response. All determinations were repeated in triplicate.

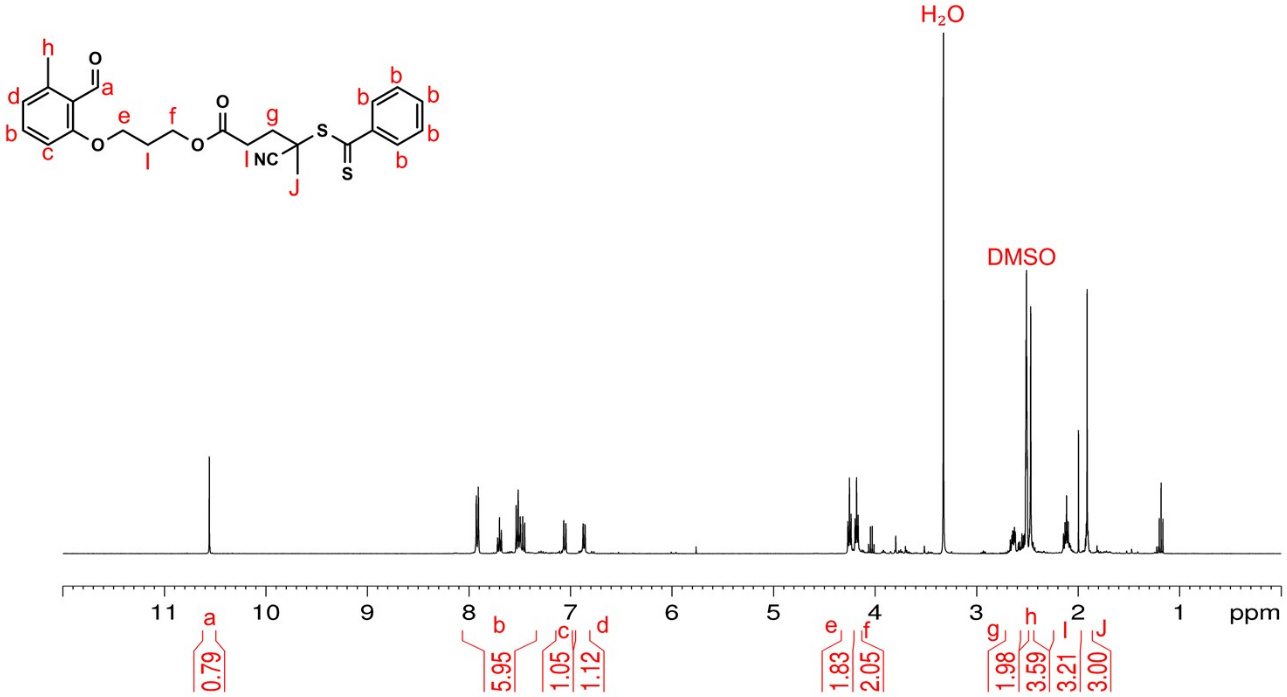

Figure S2. ^{1}H NMR spectrum of synthesized RAFT agent recorded in DMSO-d6.

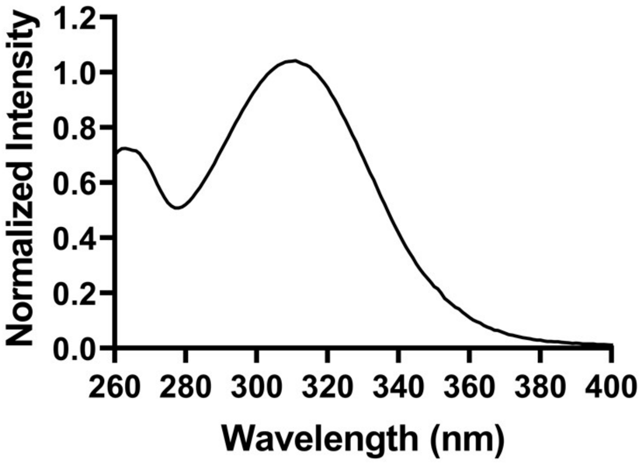

Figure S3. UV–vis spectra of RAFT agent in DMSO.

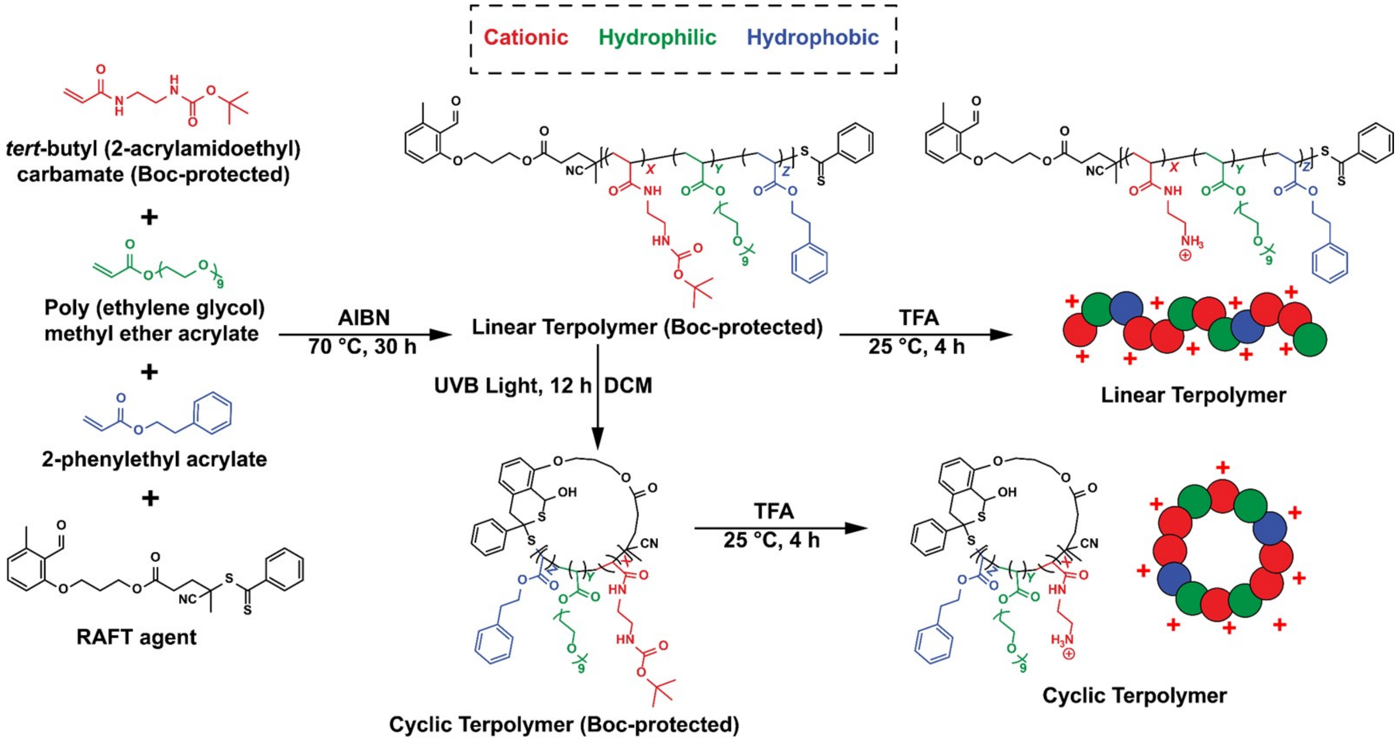

Figure S4. Synthesis scheme of linear and cyclic terpolymer. Similar to (P) group of polymers (LPP and CPP), (E) group of polymers (LPE and CPE) can also be synthesized using the represented scheme.

***Notes*:** *X* = percentage of cationic groups, *Y* = percentage of hydrophilic groups, and *Z* = percentage of hydrophobic groups. TFA: trifluoroacetic acid. AIBN: 2,2′-azobis(2-methylpropionitrile). DCM: dichloromethane.

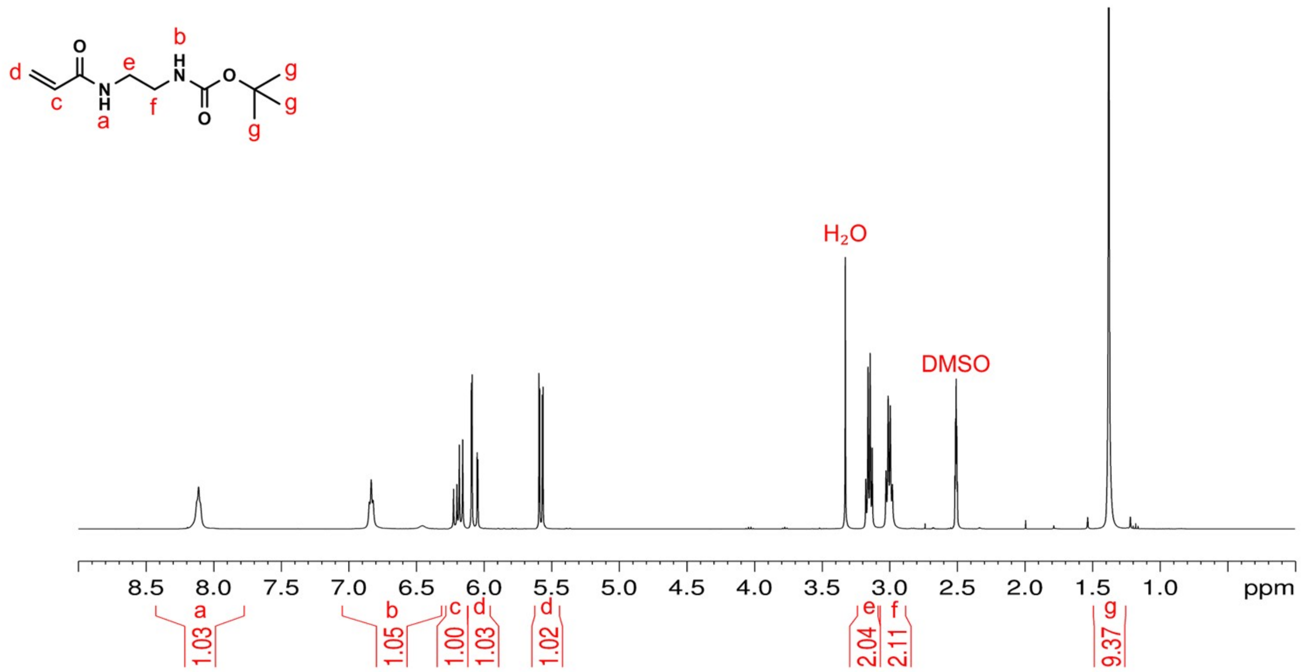

Figure S5. ^{1}H NMR spectrum of *t*ert-butyl (2-acrylamidoethyl) carbamate (cationic) monomer recorded in DMSO-d6.

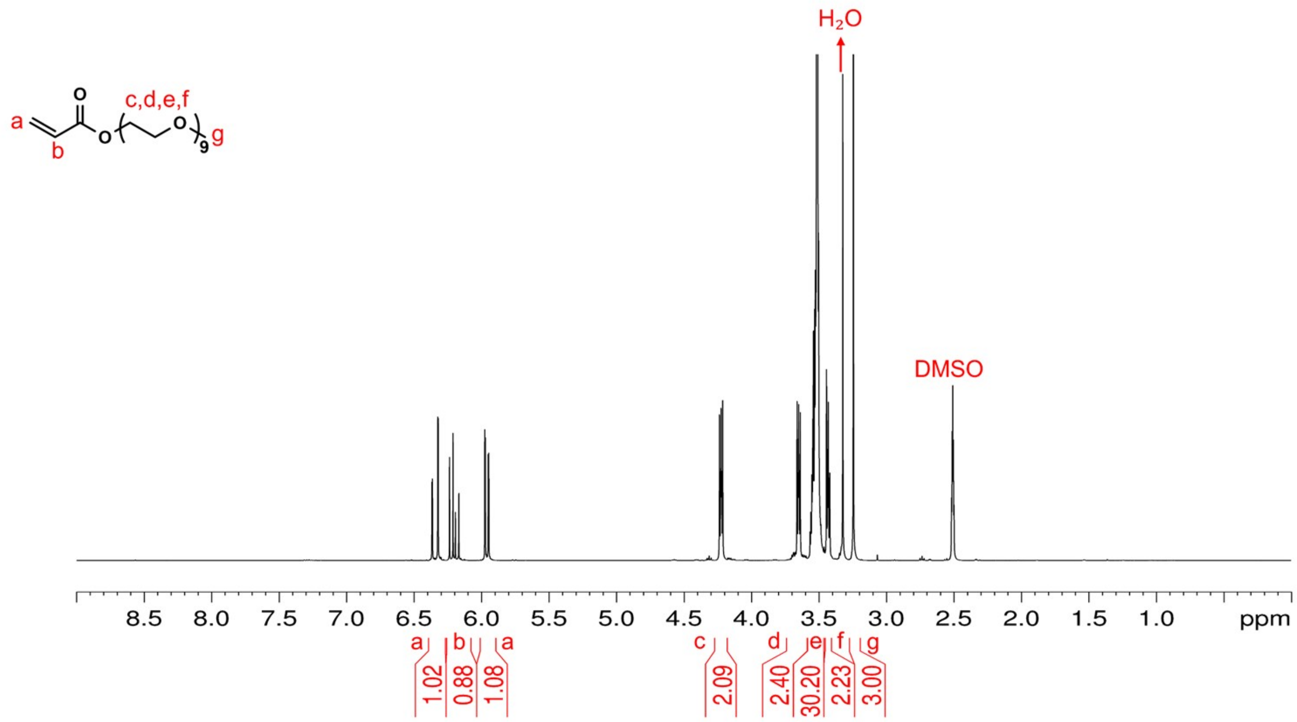

Figure S6. ^{1}H NMR spectrum of poly(ethylene glycol) methyl ether acrylate (neutral hydrophilic) monomer recorded in DMSO-d6.

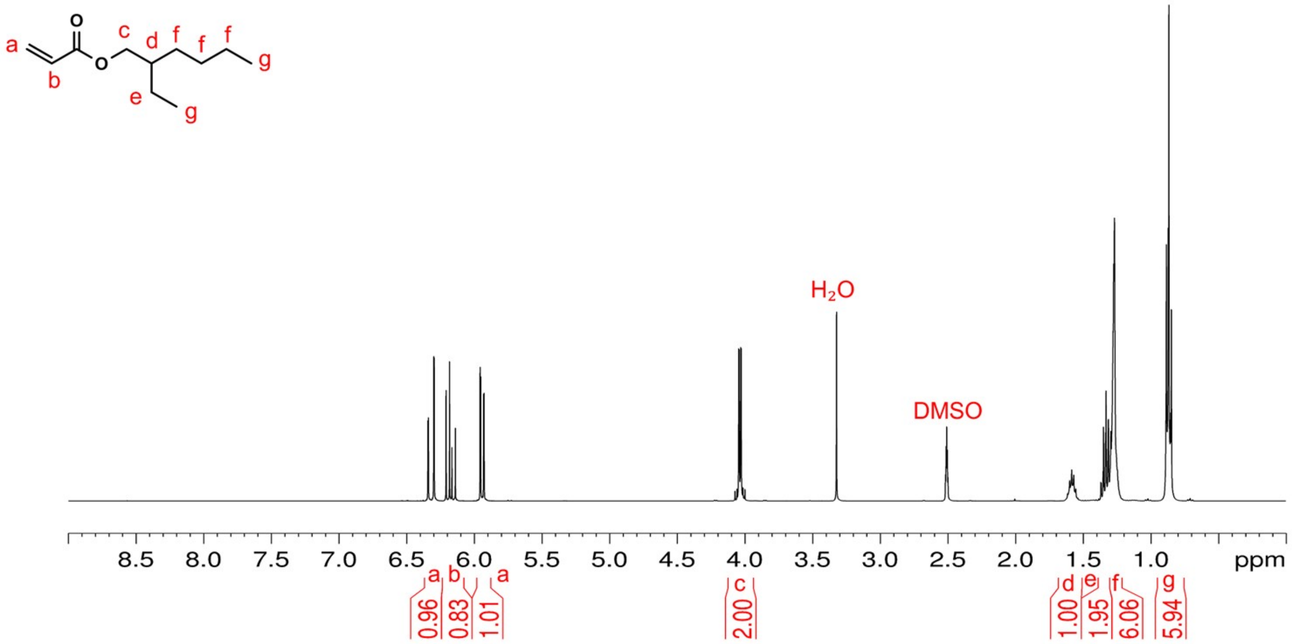

Figure S7. ^{1}H NMR spectrum of 2-ethylhexyl acrylate (hydrophobic) monomer recorded in DMSO-d6.

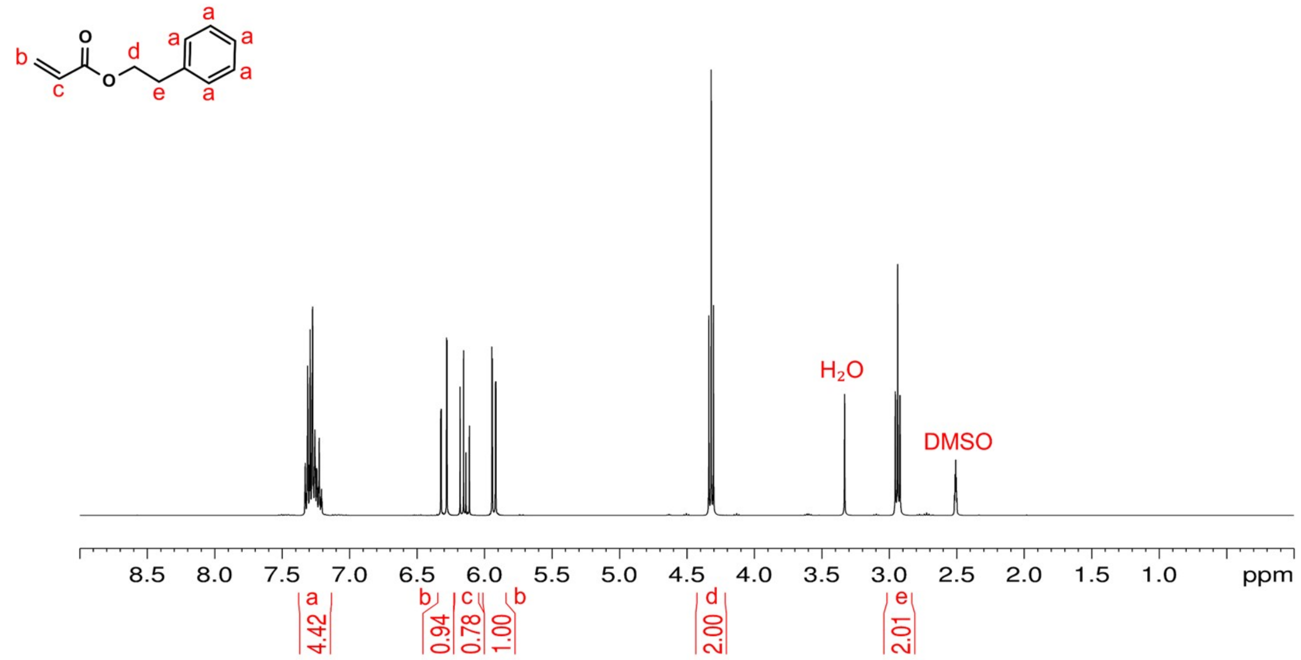

Figure S8. ^{1}H NMR spectrum of 2-phenylethyl acrylate (hydrophobic) monomer recorded in DMSO-d6.

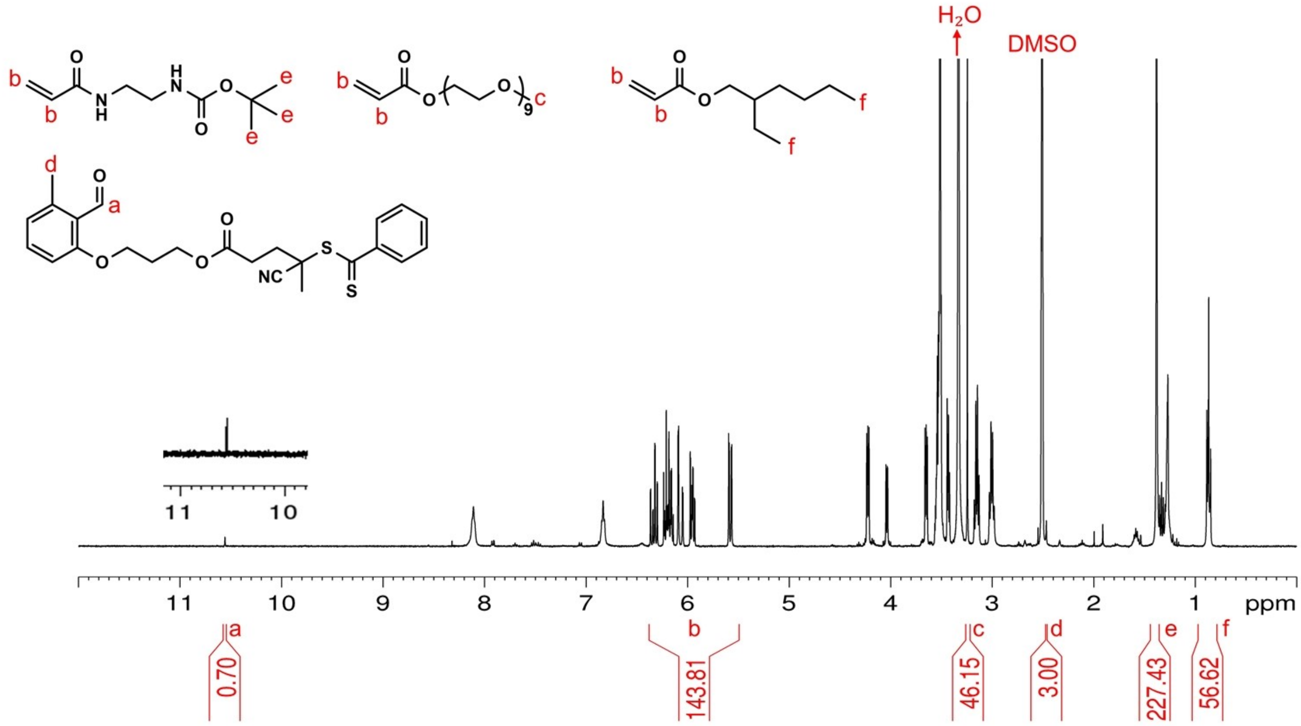

Figure S9. ^{1}H NMR spectrum of reaction mixture used for the preparation of Boc-protected linear terpolymer (LPE-20) at t = 0 recorded in DMSO-d6. Peak labels a−f correspond to the specific assignments of the signals to the RAFT agent and monomer structures and were used for composition calculations.

$$\% \text{ Cationic monomer} = \frac{\frac{\int e}{9}}{\frac{\int e}{9}+\frac{\int c}{3}+\frac{\int f}{6}} = \frac{\frac{227.43}{9}}{\frac{227.43}{9}+\frac{46.15}{3}+\frac{56.62}{6}} \times 100\% = 50.4\%$$

$$\% \text{ Hydrophilic monomer} = \frac{\frac{\int c}{3}}{\frac{\int e}{9}+\frac{\int c}{3}+\frac{\int f}{6}} = \frac{\frac{46.15}{3}}{\frac{227.43}{9}+\frac{46.15}{3}+\frac{56.62}{6}} \times 100\% = 30.7\%$$

$$\% \text{ 2-ethylhexyl monomer} = \frac{\frac{\int f}{6}}{\frac{\int e}{9}+\frac{\int c}{3}+\frac{\int f}{6}} = \frac{\frac{56.62}{6}}{\frac{227.43}{9}+\frac{46.15}{3}+\frac{56.62}{6}} \times 100\% = 18.8\%$$

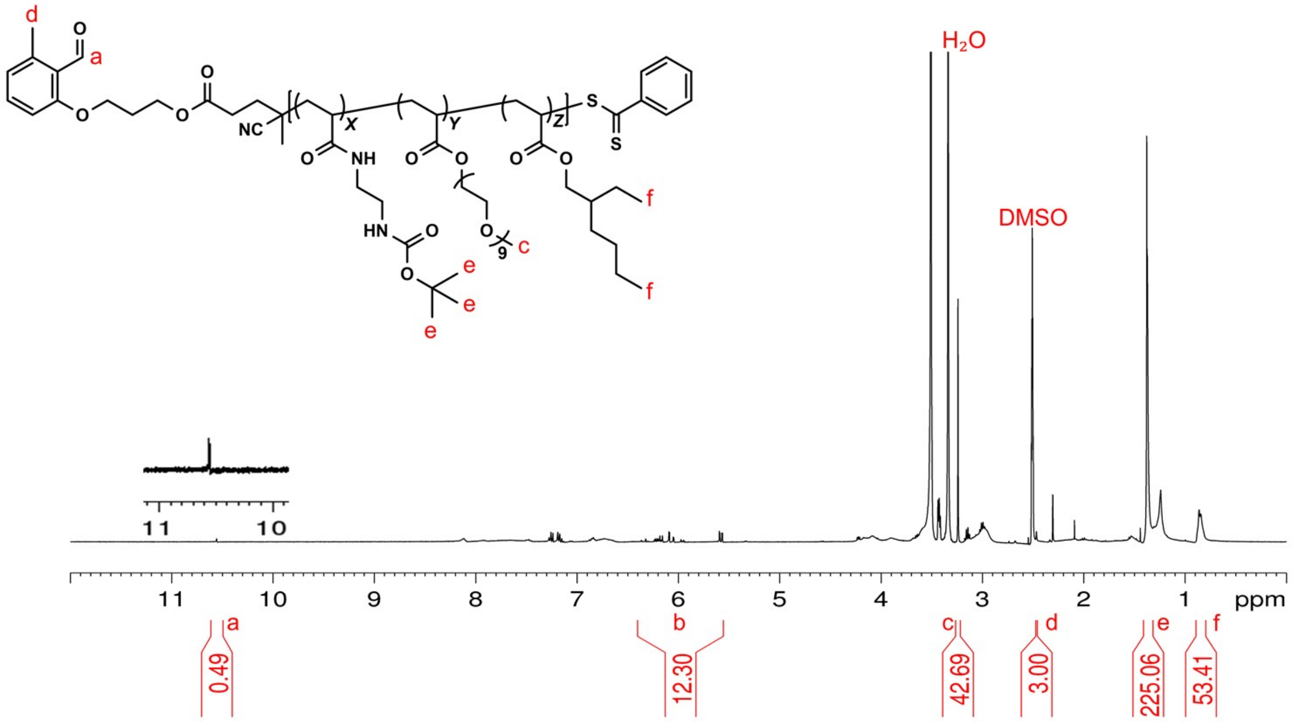

Figure S10. ^{1}H NMR spectrum of Boc-protected LPE-20 after 30 h polymerization in DMSO-d6 before purification. Peak labels a–f correspond to the specific assignments of the signals to the RAFT agent and terpolymer structure and were used for composition calculations. Peak label (b) correspond to the residual vinyl signals of the cationic, hydrophilic and 2-ethylhexyl monomers and were normalised by (d) (based on the RAFT signal, see **Figure S9**) to calculate the monomer conversion (%).

$$\% \text{ Cationic monomer} = \frac{\frac{\int e}{9}}{\frac{\int e}{9}+\frac{\int c}{3}+\frac{\int f}{6}} = \frac{\frac{225.06}{9}}{\frac{225.06}{9}+\frac{42.69}{3}+\frac{53.41}{6}} \times 100\% = 51.9\%$$

$$\% \text{ Hydrophilic monomer} = \frac{\frac{\int c}{3}}{\frac{\int e}{9}+\frac{\int c}{3}+\frac{\int f}{6}} = \frac{\frac{42.69}{3}}{\frac{225.06}{9}+\frac{42.69}{3}+\frac{53.41}{6}} \times 100\% = 29.5\%$$

$$\% \text{ 2-ethylhexyl monomer} = \frac{\frac{\int f}{6}}{\frac{\int e}{9}+\frac{\int c}{3}+\frac{\int f}{6}} = \frac{\frac{53.41}{6}}{\frac{225.06}{9}+\frac{42.69}{3}+\frac{53.41}{6}} \times 100\% = 18.4\%$$

$$\% \text{ Monomer conversion} = 100\% - \left(\frac{\int b\ polymer}{\int b_{\ t0}}\right) \times 100\% = 100\% - \left(\frac{12.30}{143.81}\right) \times 100\%$$

$$= 100\% - 8.55\% = 91.4\%$$

Notes**:** After polymerization, the low vinyl signals at δ 5.6–6.6 ppm indicates monomer conversion of 91.4%. X = percentage of cationic groups, Y = percentage of hydrophilic groups, and Z = percentage of hydrophobic groups.

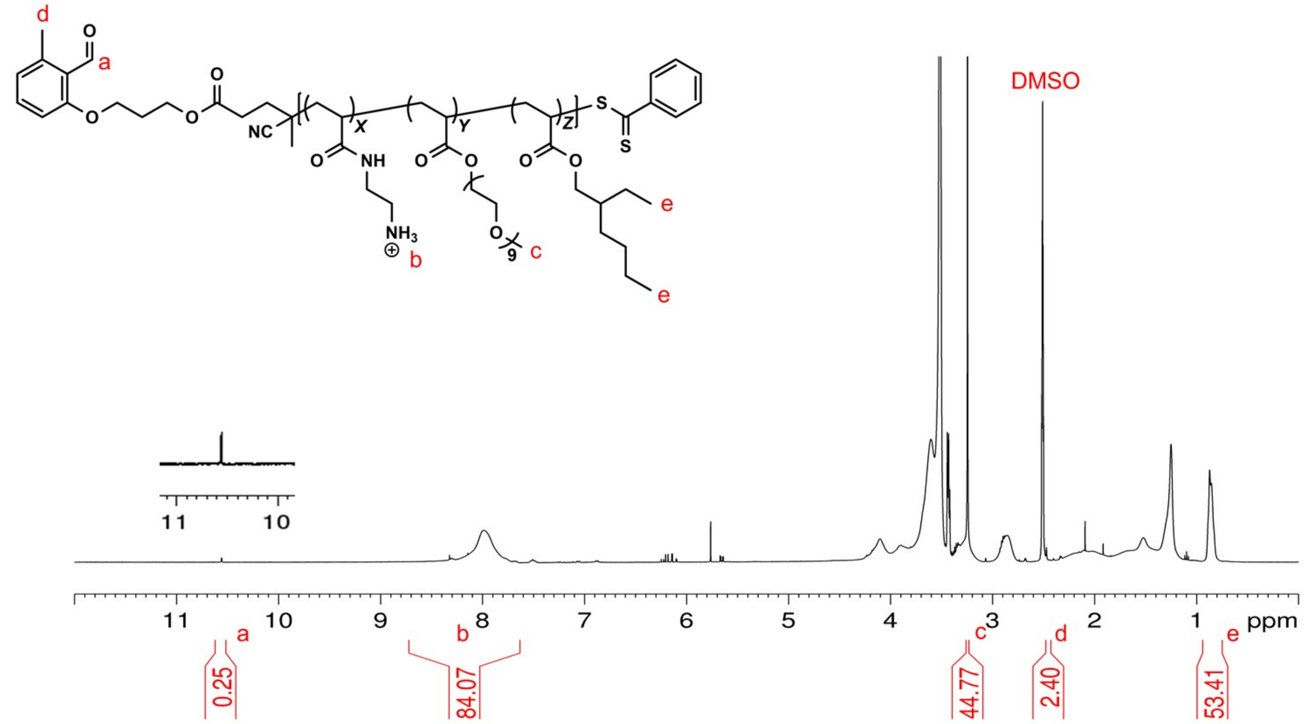

Figure S11. ^{1}H NMR spectrum of deprotected LPE-20 in DMSO-d6. a, b, c, d, and e correspond to the specific assignments of the signals to the RAFT agent and terpolymer structure. Signal e (2-ethylhexyl monomer) was set from the integral value obtained in signal f (based on the hydrophobic monomer signal, see **Figure S10**).

***Notes*:** The absence of Boc-group peak signal at around 1.4 ppm confirms its successful removal. *X* = percentage of cationic groups, *Y* = percentage of hydrophilic groups, and *Z* = percentage of hydrophobic groups.

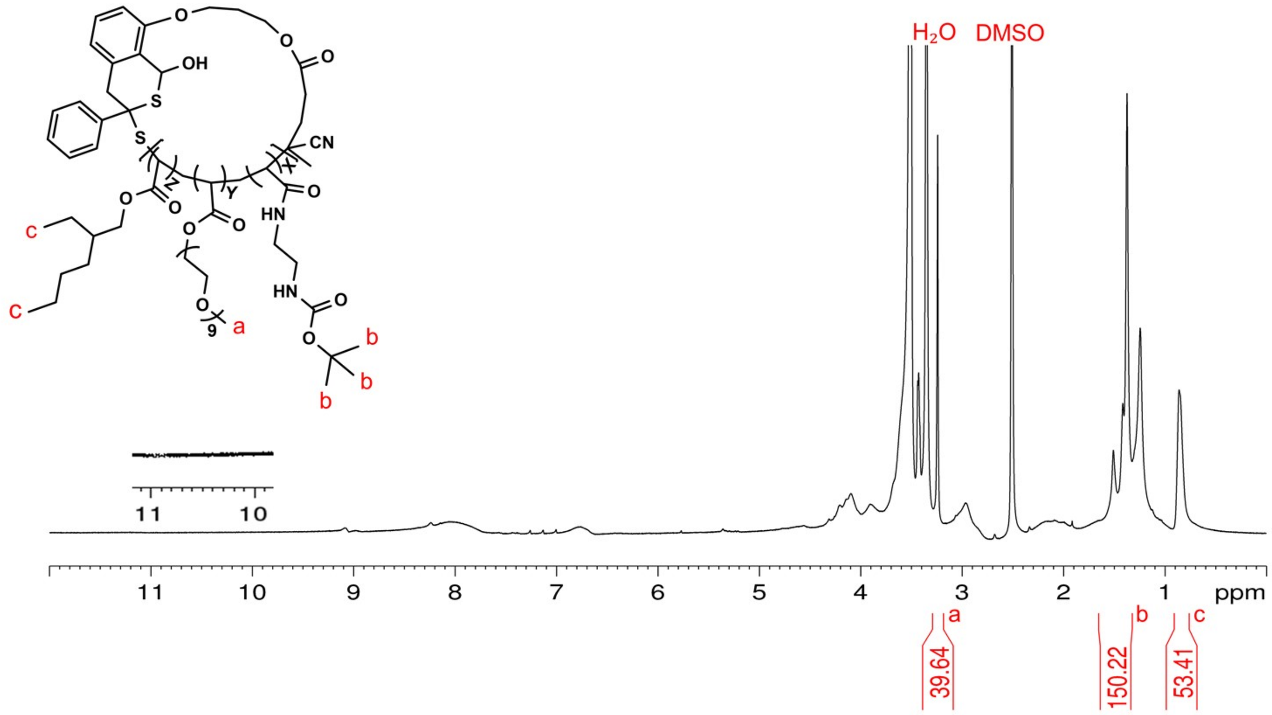

Figure S12. ^{1}H NMR spectrum of Boc-protected CPE-20 after cyclization in DMSO-d6. Peak labels a, b, and c correspond to the specific assignments of the signals to the cyclic polymer structure. Signal c (2-ethylhexyl

monomer) was set from the integral value obtained in signal f (based on the hydrophobic monomer signal, see **Figure S10**).

***Notes*:** The ^{1}H-NMR spectrum of Boc-protected CPE-20 indicated the complete disappearance of peaks at around 10.55 and 2.46 ppm, which correspond to the orthoquinodimethane end group of the Boc-protected linear precursors (see **Figure S10**).[3, 14] However, due to the complexity of our polymerization system (involving ternary monomers), the proton of the newly formed isothiochroman group could not be identified in the ^{1}H-NMR spectrum. *X* = percentage of cationic groups, *Y* = percentage of hydrophilic groups, and *Z* = percentage of hydrophobic groups.

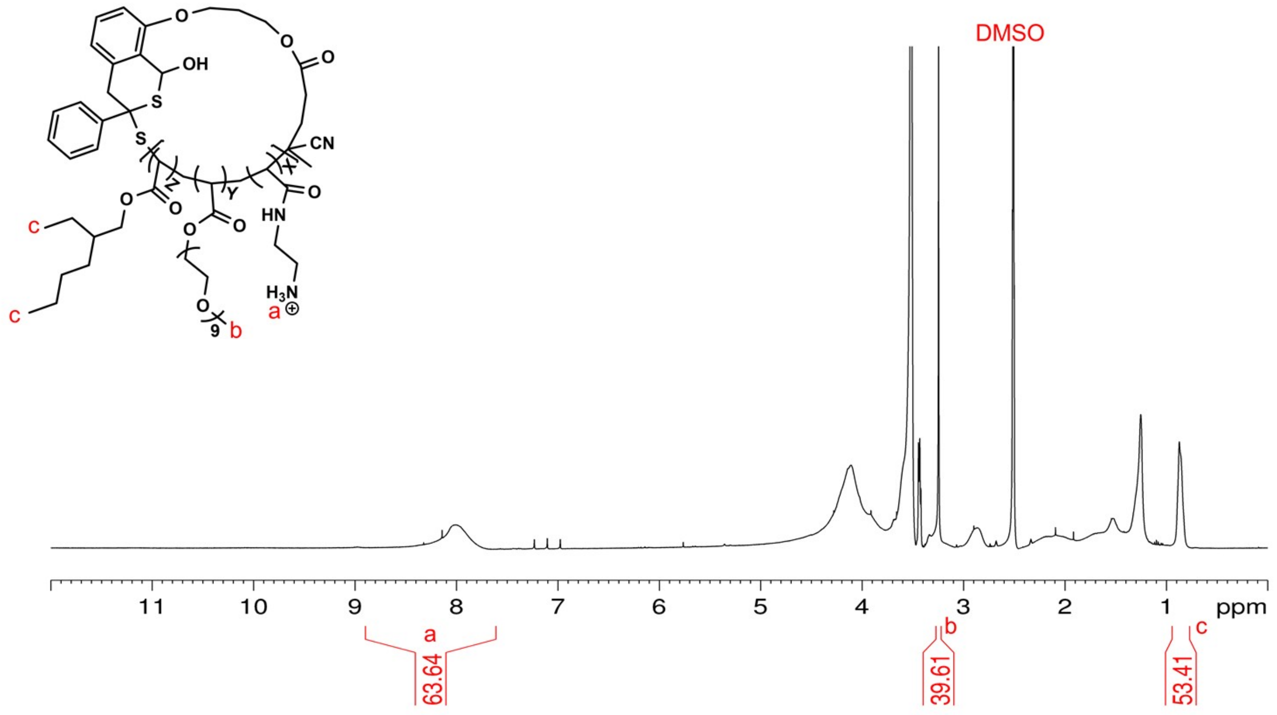

Figure S13. ^{1}H NMR spectrum of deprotected CPE-20 in DMSO-d6. a, b, and c correspond to the specific assignments of the signals to the terpolymer structure.

***Notes*:** The absence of Boc-group peak signal at around 1.4 ppm confirms its successful removal. *X* = percentage of cationic groups, *Y* = percentage of hydrophilic groups, and *Z* = percentage of hydrophobic groups.

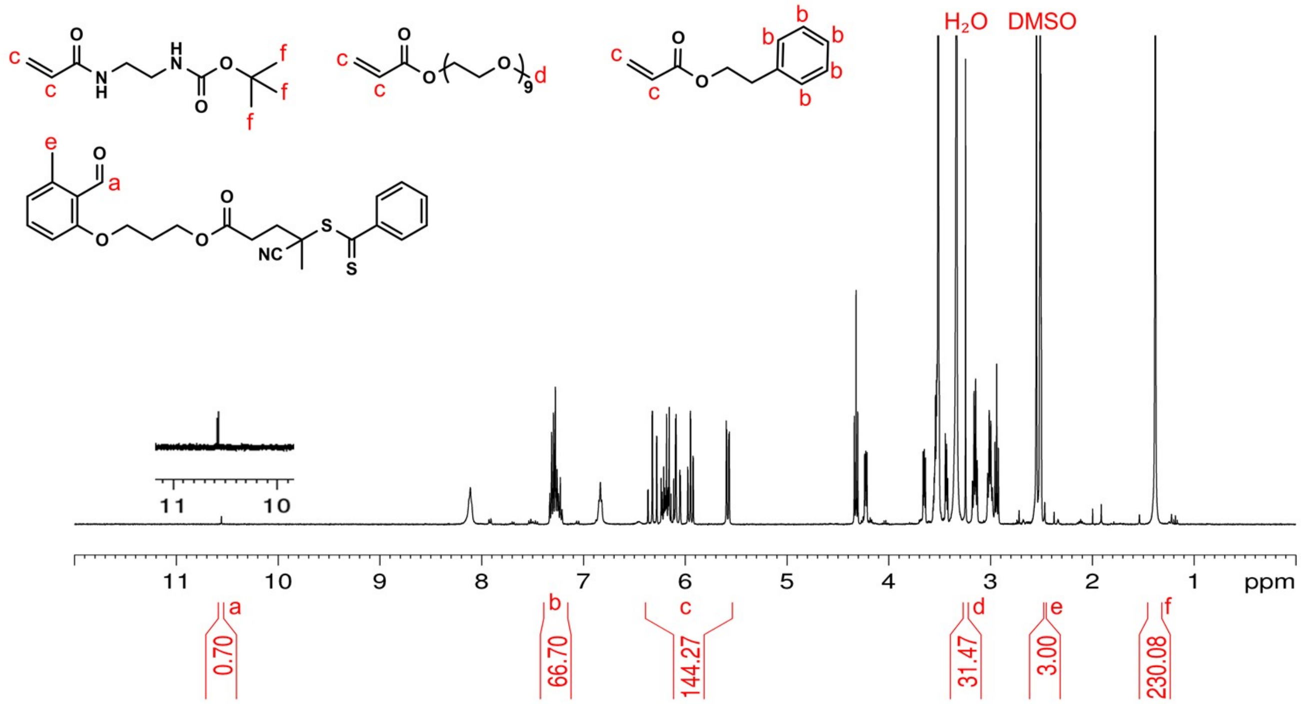

Figure S14. ^{1}H NMR spectrum of reaction mixture used for the preparation of Boc-protected linear terpolymer (LPP-30) at t = 0 recorded in DMSO-d6. Peak labels a−f correspond to the specific assignments of the signals to the RAFT agent and monomer structures and were used for composition calculations.

$$\% \text{ Cationic monomer} = \frac{\frac{\int f}{9}}{\frac{\int f}{9}+\frac{\int d}{3}+\frac{\int b}{5}} = \frac{\frac{230.08}{9}}{\frac{230.08}{9}+\frac{31.47}{3}+\frac{66.70}{5}} \times 100\% = 51.7\%$$

$$\% \text{ Hydrophilic monomer} = \frac{\frac{\int d}{3}}{\frac{\int f}{9}+\frac{\int d}{3}+\frac{\int b}{5}} = \frac{\frac{31.47}{3}}{\frac{230.08}{9}+\frac{31.47}{3}+\frac{66.70}{5}} \times 100\% = 21.2\%$$

$$\% \text{ 2-phenylethyl monomer} = \frac{\frac{\int b}{5}}{\frac{\int f}{9}+\frac{\int d}{3}+\frac{\int b}{5}} = \frac{\frac{66.70}{5}}{\frac{230.08}{9}+\frac{31.47}{3}+\frac{66.70}{5}} \times 100\% = 27.0\%$$

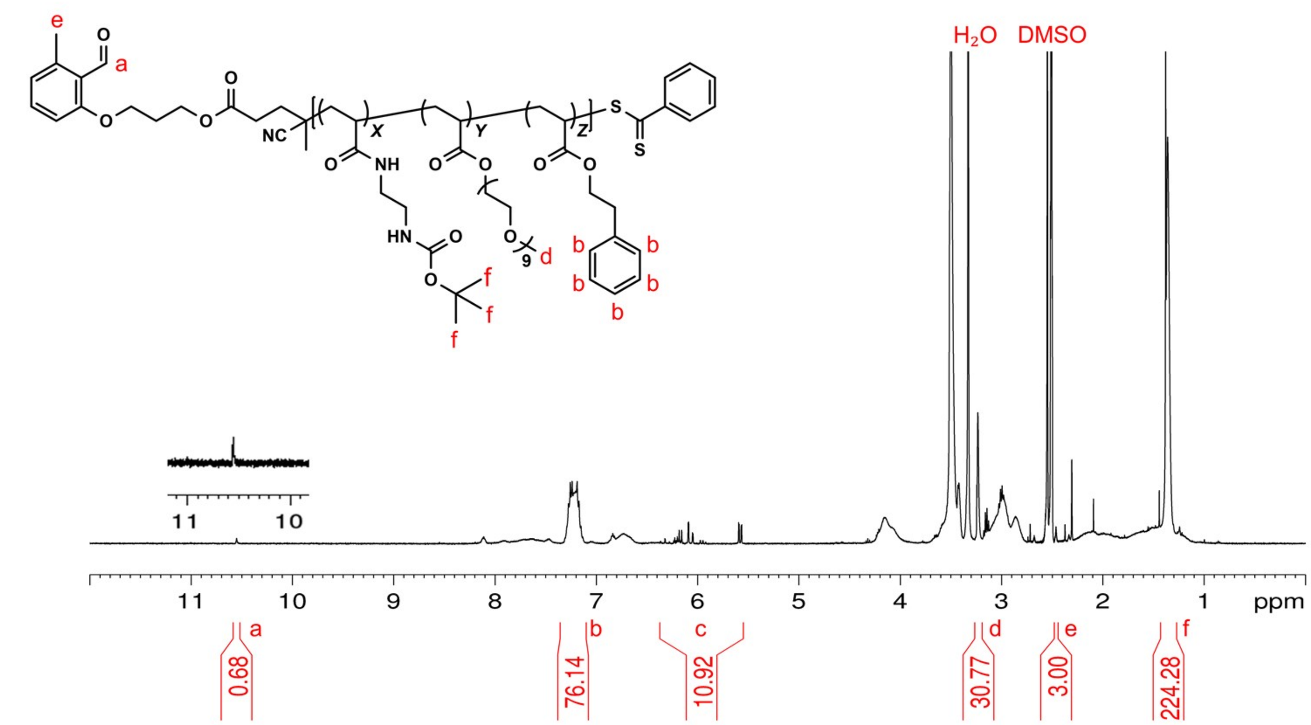

Figure S15. ^{1}H NMR spectrum of Boc-protected LPP-30 after 30 h polymerization in DMSO-d6 before purification. Peak labels a–f correspond to the specific assignments of the signals to the RAFT agent and terpolymer structure and were used for composition calculations. Peak label (c) correspond to the residual vinyl signals of the cationic, hydrophilic and 2-phenylethyl monomers and were normalised by (e) (based on the RAFT signal, see **Figure S14**) to calculate the monomer conversion (%).

$$\% \text{ Cationic monomer} = \frac{\frac{\int f}{9}}{\frac{\int f}{9}+\frac{\int d}{3}+\frac{\int b}{5}} = \frac{\frac{224.28}{9}}{\frac{224.28}{9}+\frac{30.77}{3}+\frac{76.14}{5}} \times 100\% = 49.4\%$$

$$\% \text{ Hydrophilic monomer} = \frac{\frac{\int d}{3}}{\frac{\int f}{9}+\frac{\int d}{3}+\frac{\int b}{5}} = \frac{\frac{30.77}{3}}{\frac{224.28}{9}+\frac{30.77}{3}+\frac{76.14}{5}} \times 100\% = 20.3\%$$

$$\% \text{ 2-phenylethyl monomer} = \frac{\frac{\int b}{5}}{\frac{\int f}{9}+\frac{\int d}{3}+\frac{\int b}{5}} = \frac{\frac{76.14}{5}}{\frac{224.28}{9}+\frac{30.77}{3}+\frac{76.14}{5}} \times 100\% = 30.2\%$$

$$\% \text{ Monomer conversion} = 100\% - \left(\frac{\int c\, polymer}{\int c_{t0}}\right) \times 100\% = 100\% - \left(\frac{10.92}{144.26}\right) \times 100\%$$

$$= 100\% - 7.56\% = 92.4\%$$

Notes**:** After polymerization, the low vinyl signals at δ 5.5–6.3 ppm indicates monomer conversion of 92.4%. X = percentage of cationic groups, Y = percentage of hydrophilic groups, and Z = percentage of hydrophobic groups.

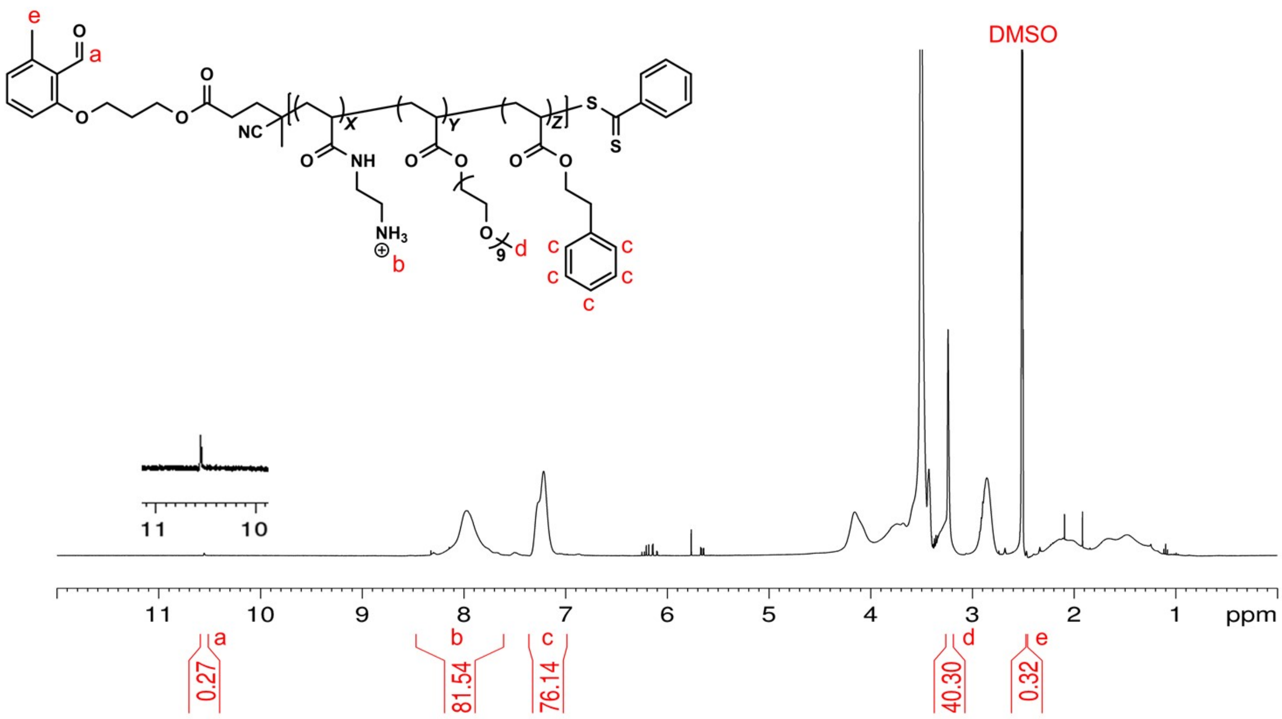

Figure S16. ^{1}H NMR spectrum of deprotected LPP-30 in DMSO-d6. a, b, c, d, and e correspond to the specific assignments of the signals to the RAFT agent and linear terpolymer structure. Signal c (2-phenylethyl monomer) was set from the integral value obtained in signal b (based on the hydrophobic monomer signal, see **Figure S15**).

Notes**:** The absence of Boc-group peak signal at around 1.4 ppm confirms its successful removal. X = percentage of cationic groups, Y = percentage of hydrophilic groups, and Z = percentage of hydrophobic groups.

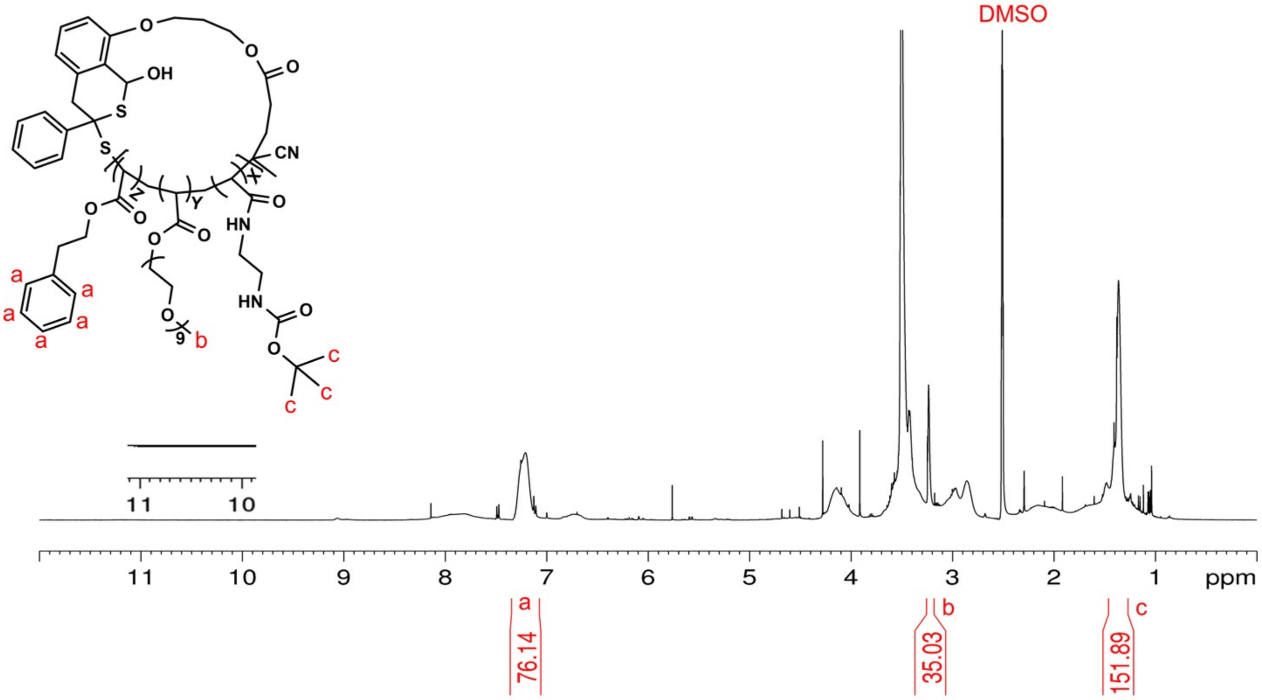

Figure S17. ^{1}H NMR spectrum of Boc-protected CPP-30 after cyclization in DMSO-d6. Peak labels a, b, and c correspond to the specific assignments of the signals to the cyclic polymer structure. Signal a (2-phenylethyl monomer) was set from the integral value obtained in signal b (based on the hydrophobic monomer signal, see **Figure S15**).

Notes**:** The ^{1}H-NMR spectrum of Boc-protected CPE-20 indicated the complete disappearance of peaks at around 10.55 and 2.46 ppm, which correspond to the orthoquinodimethane end group of the Boc-protected linear precursors (see **Figure S15**).[3, 14] However, due to the complexity of our polymerization system (involving ternary monomers), the proton of the newly formed isothiochroman group could not be identified in the ^{1}H-NMR spectrum. *X* = percentage of cationic groups, *Y* = percentage of hydrophilic groups, and *Z* = percentage of hydrophobic groups.

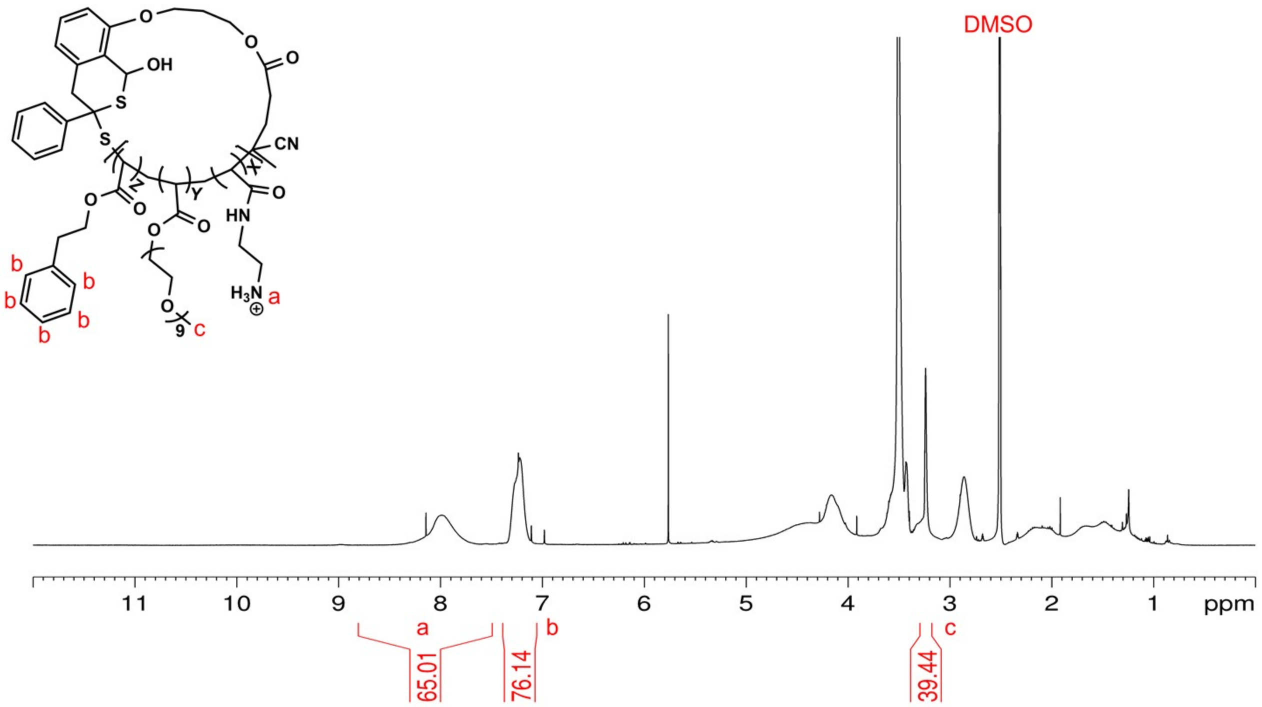

Figure S18. ^1H NMR spectrum of deprotected CPP-30 in DMSO-d6. a, b, and c correspond to the specific assignments of the signals to the cyclic terpolymer structure.

Notes**:** The absence of Boc-group peak signal at around 1.4 ppm confirms its successful removal. X = percentage of cationic groups, Y = percentage of hydrophilic groups, and Z = percentage of hydrophobic groups.

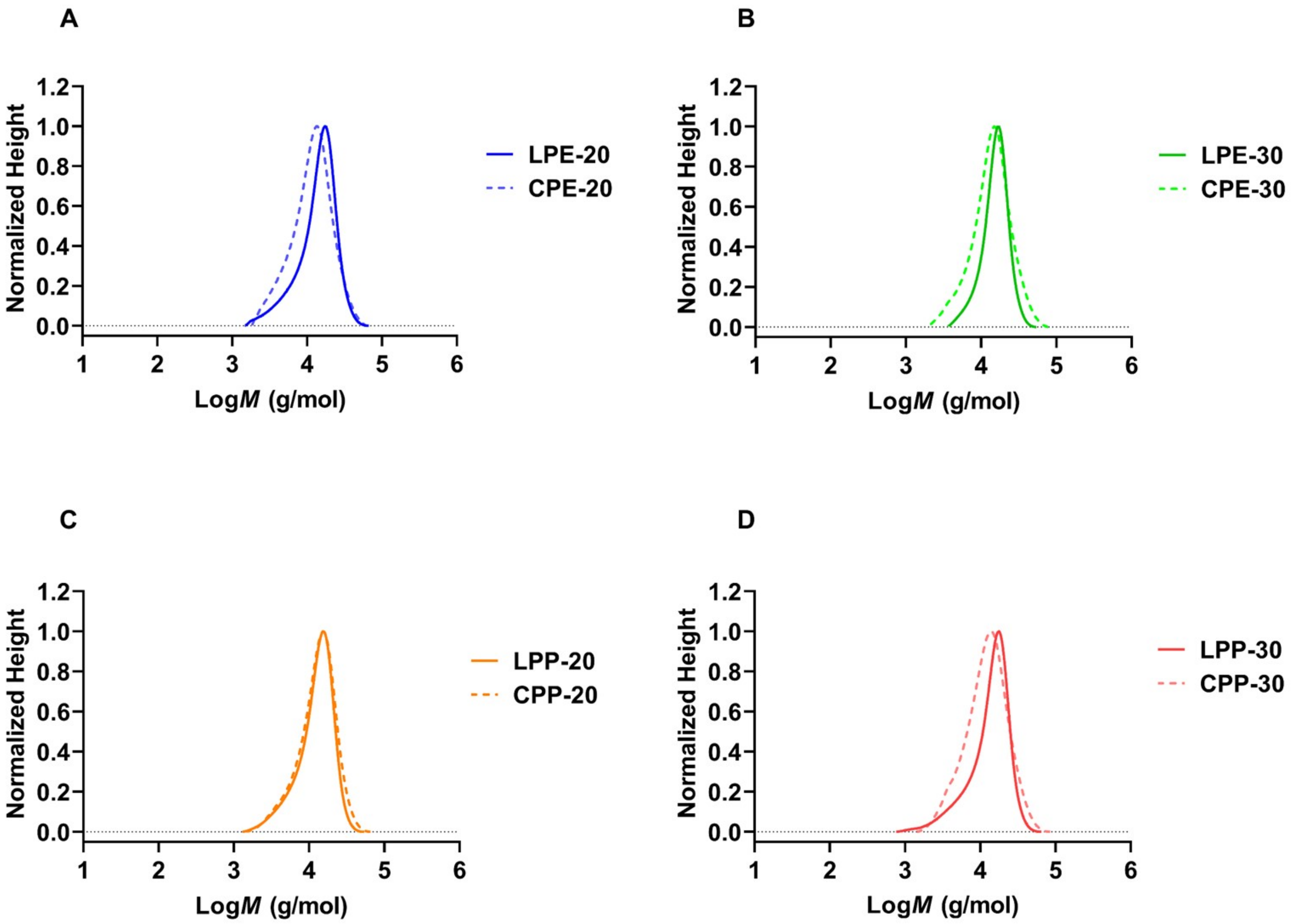

Figure S19. Molecular weight distributions of the Boc-protected terpolymers: (A) LPE-20 and CPE-20, (B) LPE-30 and CPE-30, (C) LPP-20 and CPP-20, and (D) LPP-30 and CPP-30 obtained by size-exclusion chromatography (SEC).

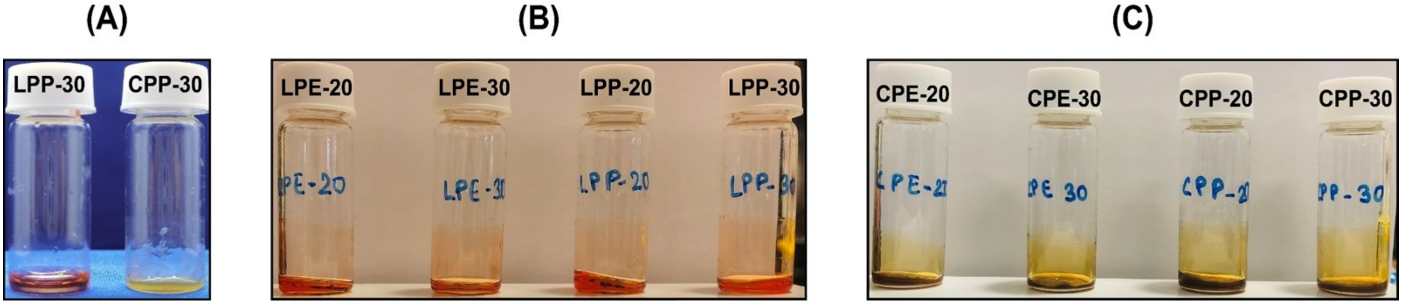

Figure S20. (A) Photos of Boc-protected LP (LPP-30) and its corresponding CP (CPP-30). (B) and (C) are photos of deprotected LPs (LPE-20, LPE-30, LPP-20, and LPP-30) and their resultant CPs (CPE-20, CPE-30, CPP-20, and CPP-30).

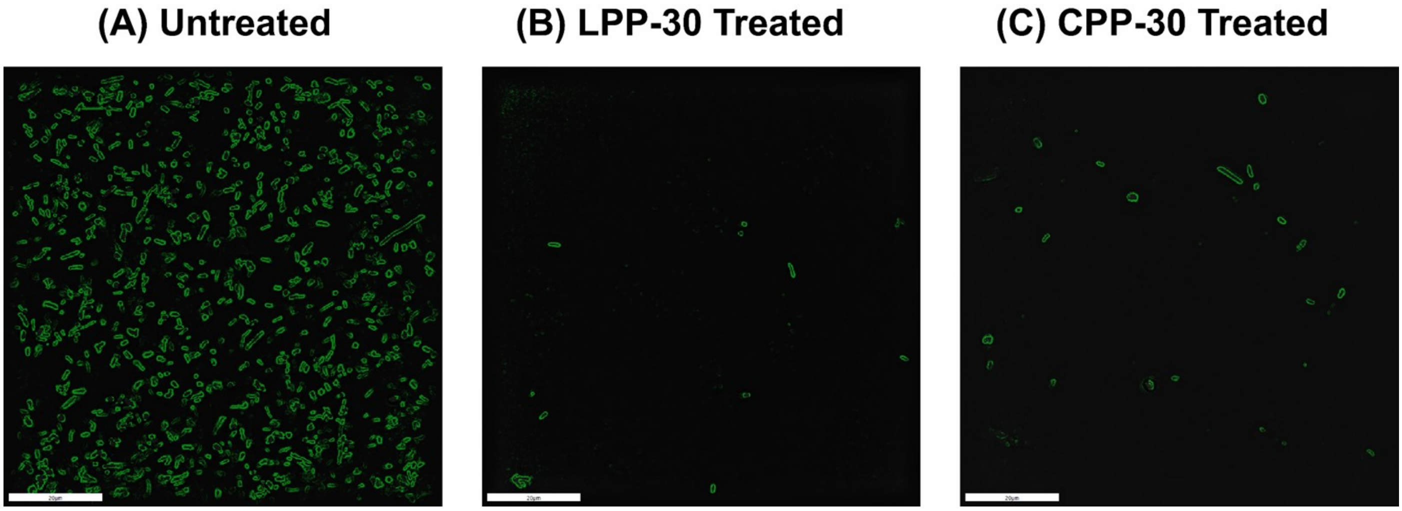

Figure S21. 2D tomographic microscopy images of the untreated control (EC K12) (A), MIC_{90} concentration of LPP-30 and CPP-30 treated samples (B and C). MIC_{90} concentration of LPP-30 and CPP-30 were given in **Table 2.** The staining was performed using 3D tomographic microscope (3D Cell Explorer, NanoLive) equipped with digital staining software. Scale bar = 20 µm.

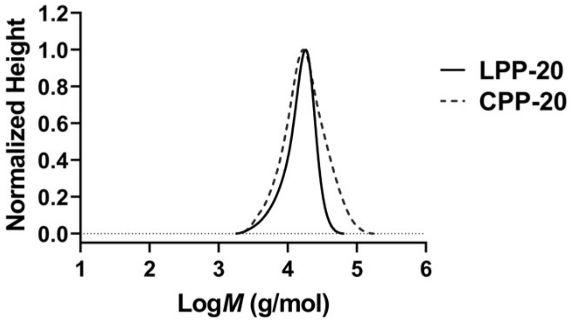

Figure S22. Molecular weight distributions of the Boc-protected LPP-20 and CPP-20 terpolymers obtained by SEC (when cyclization reaction was performed at 40 mg/mL).

**Polymer**	M_n **(kg/mol)** [a,b]	*Đ* [a,b]
**LPP-20**	13.0	1.26
**CPP-20**	14.7	1.53

Notes**:** High concentrations of the linear precursor (40 mg/mL) used in the cyclization reaction resulted in increased dispersity (*Đ*), likely due to coupling reactions and the formation of linear multiblock impurities. These factors contributed to both a higher number-average molecular weight (M_n) and *Đ*. [a] Based on Boc protected terpolymers. [b] Determined by SEC-DMAc analysis using poly(methyl methacrylate) as the standard.

Table S1. Overview of monomer conversion (%) into polymer after 30 h of RAFT polymerization.

Polymer	Monomer conversion (%)[a]
**LPE-20**	91
**CPE-20**	91
**LPE-30**	93
**CPE-30**	93
**LPP-20**	90
**CPP-20**	90
**LPP-30**	92
**CPP-30**	92

Note: [a] Based on ^{1}H NMR spectroscopy after polymerization.

Table S2. Average hydrodynamic diameter (D_h) and zeta potential (ζ) of terpolymers in PBS (pH 7.4) were measured using DLS analysis.

Polymer	D_h* (nm)	ζ* (mV)
**LPE-20**	4.3±0.7	12.6±1.1
**CPE-20**	5.0±0.3	10.5±0.4
**LPE-30**	3.6±0.8	14.7±1.6
**CPE-30**	5.3±0.6	13.0±1.5
**LPP-20**	4.1±0.2	13.4±2.2
**CPP-20**	6.4±0.4	12.2±0.4
**LPP-30**	8.0±1.4	7.1±0.3
**CPP-30**	5.3±1.0	13.1±1.1

Note: * Based on Boc-deprotected polymers.

Table S3. Size distribution by intensity and volume in Milli-Q water and PBS (pH 7.4), measured using DLS analysis.

Polymer	Milli-Q water		PBS (pH 7.4)	
	**Size distribution by intensity (nm)**	**Size distribution by volume (nm)**	**Size distribution by intensity (nm)**	**Size distribution by volume (nm)**
**LPE-20**	197.6±0	219.0±15.1	209.8±28.2	4.8±0.6
**CPE-20**	179.1±13.0	167.3±42.2	161.9±11.2	5.8±0.4
**LPE-30**	229.8±0	229.8±0	189.8±28.2	5.0±0.3
**CPE-30**	219.0±15.1	242.2±17.6	197.6±0	6.1±0.7
**LPP-20**	267.2±0	254.7±17.6	219.0±15.1	4.5±0
**CPP-20**	208.3±15.1	219.0±15.1	188.3±13.0	6.7±0.4
**LPP-30**	229.8±0	242.2±17.6	161.9±11.2	9.0±2.1
**CPP-30**	229.8±0	242.2±17.6	188.3±13.0	5.9±1.0

Note: * Based on Boc-deprotected polymers.

References

1. K. K. Oehlenschlaeger, J. O. Mueller, N. B. Heine, M. Glassner, N. K. Guimard, G. Delaittre, F. G. Schmidt and C. Barner-Kowollik, Light-induced modular ligation of conventional RAFT polymers, *Angewandte Chemie International Edition*, 2013, **52**.
2. T.-K. Nguyen, S. J. Lam, K. K. Ho, N. Kumar, G. G. Qiao, S. Egan, C. Boyer and E. H. Wong, Rational design of single-chain polymeric nanoparticles that kill planktonic and biofilm bacteria, *ACS Infectious Diseases*, 2017, **3**, 237-248.
3. Q. Tang, Y. Wu, P. Sun, Y. Chen and K. Zhang, Powerful ring-closure method for preparing varied cyclic polymers, *Macromolecules*, 2014, **47**, 3775-3781.
4. P. R. Judzewitsch, L. Zhao, E. H. Wong and C. Boyer, High-throughput synthesis of antimicrobial copolymers and rapid evaluation of their bioactivity, *Macromolecules*, 2019, **52**, 3975-3986.
5. R. Namivandi-Zangeneh, Z. Sadrearhami, D. Dutta, M. Willcox, E. H. Wong and C. Boyer, Synergy between synthetic antimicrobial polymer and antibiotics: a promising platform to combat multidrug-resistant bacteria, *ACS infectious diseases*, 2019, **5**, 1357-1365.
6. D. Dutta, A. K. Vijay, N. Kumar and M. D. Willcox, Melimine-coated antimicrobial contact lenses reduce microbial keratitis in an animal model, *Investigative ophthalmology & visual science*, 2016, **57**, 5616-5624.
7. D. Dutta, N. Cole, N. Kumar and M. D. Willcox, Broad spectrum antimicrobial activity of melimine covalently bound to contact lenses, *Investigative ophthalmology & visual science*, 2013, **54**, 175-182.
8. M. A. Hudson, D. A. Siegele and S. W. Lockless, Use of a fluorescence-based assay to measure Escherichia coli membrane potential changes in high throughput, *Antimicrobial agents and chemotherapy*, 2020, **64**, 10.1128/aac. 00910-00920.
9. T. Zhang, W. An, J. Sun, F. Duan, Z. Shao, F. Zhang, T. Jiang, X. Deng, C. Boyer and W. Gao, N-terminal lysozyme conjugation to a cationic polymer enhances antimicrobial activity and overcomes antimicrobial resistance, *Nano Letters*, 2022, **22**, 8294-8303.
10. Z. Shao, K. Hakobyan, J. Xu, R. Chen, N. Kumar, M. Willcox and E. H. Wong, Photoinduced Unveiling of Cationic Amine: Toward Smart Photoresponsive Antimicrobial Polymers, *ACS Applied Polymer Materials*, 2023, **5**, 8735-8743.

11. P. R. Judzewitsch, T. K. Nguyen, S. Shanmugam, E. H. Wong and C. Boyer, Towards sequence-controlled antimicrobial polymers: effect of polymer block order on antimicrobial activity, *Angewandte Chemie International Edition*, 2018, **57**, 4559-4564.
12. R. Namivandi-Zangeneh, R. J. Kwan, T.-K. Nguyen, J. Yeow, F. L. Byrne, S. H. Oehlers, E. H. Wong and C. Boyer, The effects of polymer topology and chain length on the antimicrobial activity and hemocompatibility of amphiphilic ternary copolymers, *Polymer Chemistry*, 2018, **9**, 1735-1744.
13. Y. Gong, X. Xu, M. Aquib, Y. Zhang, W. Yang, Y. Chang, H. Peng, C. Boyer, A. K. Whittaker and C. Fu, Ammonium, Phosphonium, and Sulfonium Polymers for Antimicrobial Applications: A Comparative Study, *ACS Applied Polymer Materials*, 2024.
14. P. Sun, Z. Zhang and K. Zhang, Scalable preparation of cyclic polymers by the ring-closure method assisted by the continuous-flow technique, *Polymer Chemistry*, 2016, **7**, 2239-2244.
